# Dp412e: a novel human embryonic dystrophin isoform induced by BMP4 in early differentiated cells

**DOI:** 10.1186/s13395-015-0062-6

**Published:** 2015-11-14

**Authors:** Emmanuelle Massouridès, Jérôme Polentes, Philippe-Emmanuel Mangeot, Virginie Mournetas, Juliette Nectoux, Nathalie Deburgrave, Patrick Nusbaum, France Leturcq, Linda Popplewell, George Dickson, Nicolas Wein, Kevin M. Flanigan, Marc Peschanski, Jamel Chelly, Christian Pinset

**Affiliations:** I-STEM, CECS, Génopôle-Campus 1, 5 rue Henri Desbruères, 91030 Evry, Cedex France; CIRI, International Center for Infectiology Research, Université de Lyon, Lyon, France; Inserm, U1111, Lyon, France; CNRS, UMR5308, Lyon, France; Ecole Normale Supérieure de Lyon, Lyon, France; Université Lyon 1, Centre International de Recherche en Infectiologie, Lyon, France; UEVE U861, 91030 Evry, France; Inserm U861, 91030 Evry, France; Service de Biochimie et Génétique Moléculaire, HUPC Hôpital Cochin, Paris, France; School of Biological Sciences, Royal Holloway—University of London, Surrey, TW20 0EX UK; Center for Gene Therapy, The Research Institute at Nationwide Children’s Hospital, Columbus, OH 43205 USA; IGBMC-CNRS UMR7104/Inserm U964, 67404 Illkirch, Cedex France

**Keywords:** BMP4, Dystrophin, Duchenne muscular dystrophy, Embryonic, hiPSCs, hESCs, Exon skipping, Anthropoids, Isoform, Human

## Abstract

**Background:**

Duchenne muscular dystrophy (DMD) is a devastating X-linked recessive genetic myopathy. DMD physiopathology is still not fully understood and a prenatal onset is suspected but difficult to address.

**Methods:**

The bone morphogenetic protein 4 (BMP4) is a critical signaling molecule involved in mesoderm commitment. Human induced pluripotent stem cells (hiPSCs) from DMD and healthy individuals and human embryonic stem cells (hESCs) treated with BMP4 allowed us to model the early steps of myogenesis in normal and DMD contexts.

**Results:**

Unexpectedly, 72h following BMP4 treatment, a new long *DMD* transcript was detected in all tested hiPSCs and hESCs, at levels similar to that found in adult skeletal muscle. This novel transcript named “*Dp412e*” has a specific untranslated first exon which is conserved only in a sub-group of anthropoids including human. The corresponding novel dystrophin protein of 412-kiloDalton (kDa), characterized by an N-terminal-truncated actin-binding domain, was detected in normal BMP4-treated hiPSCs/hESCs and in embryoid bodies. Finally, using a phosphorodiamidate morpholino oligomer (PMO) targeting the *DMD* exon 53, we demonstrated the feasibility of exon skipping validation with this BMP4-inducible hiPSCs model.

**Conclusions:**

In this study, the use of hiPSCs to analyze early phases of human development in normal and DMD contexts has led to the discovery of an embryonic 412 kDa dystrophin isoform. Deciphering the regulation process(es) and the function(s) associated to this new isoform can contribute to a better understanding of the DMD physiopathology and potential developmental defects. Moreover, the simple and robust BMP4-inducible model highlighted here, providing large amount of a long *DMD* transcript and the corresponding protein in only 3 days, is already well-adapted to high-throughput and high-content screening approaches. Therefore, availability of this powerful cell platform can accelerate the development, validation and improvement of DMD genetic therapies.

**Electronic supplementary material:**

The online version of this article (doi:10.1186/s13395-015-0062-6) contains supplementary material, which is available to authorized users.

## Background

The Duchenne muscular dystrophy (DMD) gene (ENSG00000198947; MIM 300377) is located on the X chromosome spanning more than two million base pairs [1, 2]. Three promoters have been described to drive expression of full-length 427-kiloDalton (kDa) dystrophin isoforms: the muscular (dystrophin protein 427 kDa muscular (Dp427m)), the cerebral (dystrophin protein 427 kDa cerebral (Dp427c)), and the purkinje (Dp427p1/p2), each of which has a specific exon 1. Other *DMD* transcripts and isoforms are expressed due to alternative promoters and splicing with specific pattern of expression along the development [3–7].

Mutations in the *DMD* gene cause Duchenne (MIM 310200) and Becker (MIM 300376) muscular dystrophies (BMD). While the majority of DMD patients have no dystrophin, resulting in a severe phenotype, milder BMD patients are characterized by expression of dystrophin proteins abnormal in quantity and/or size. The first symptoms of DMD usually appear between the ages of 2 and 5 years [8, 9]. Progressive muscle weakness typically leads to wheelchair dependency by the age of 12 years. Historically, death occurred before age 20 due to cardiac and respiratory failure, but with improved care, life expectancy has risen well into the third decade. Until now, there is no curative treatment but there are several therapeutic approaches in progress [10–14].

Since its first description in the mid-1800s [15], DMD physiopathology is not fully understood. Interestingly, analyses of X-linked muscular dystrophy (*mdx*) mice—a dystrophin-deficient model—showed that the onset of pathology can already be observed *in utero* with abnormal myogenesis [16]. The importance of dystrophin before birth was also demonstrated in *sapje* zebrafish embryos, in which the absence of dystrophin induced muscle attachment failure [17]. Furthermore, in golden retriever muscular dystrophy dog puppies (aged 1–8 days), lesions were particularly present in the most active muscles *in utero* and during the neonatal period [18].

Histological studies on human DMD fetuses also indicated that DMD consequences already appear *in utero*, with fiber diameter variability, hyaline fibers, and increased muscle nuclear size [19–21]. In line with these studies, serum creatine kinase level is already elevated at birth in DMD newborns [9, 22]. Interestingly, when mental retardation was associated with DMD, a disorganized cellular architecture was observed in post-mortem brains [23] suggesting that dystrophin could be critical for the brain development *in utero*. Moreover, a study on skeletal muscle from presymptomatic DMD patients (aged 1.5–22 months) in which the dystrophin protein was absent showed that the gene expression profile is altered [24]. The highlighted modifications in these DMD patients mostly resemble the alterations previously described in advanced stages of the disease indicating that, even without clinical symptoms, DMD is already developing during the first months of life.

Taken together, these studies suggest that absence of dystrophin has already consequences at early stages of development*.* Due to technical and regulatory issues for the research on human embryos and fetuses, we decided to further address this question by producing human induced pluripotent stem cells (hiPSCs) [25] from healthy and DMD muscular cells. To compare the two genetic contexts during the early steps of myogenesis, we used a member of the transforming growth factor beta (TGF-β) superfamily, bone morphogenetic protein 4 (BMP4) involved in mesoderm commitment [26–29]. Interestingly, BMP4 induced the expression of a long *DMD* transcript in early mesoderm precursors derived from either DMD/normal hiPSCs or normal human embryonic stem cells (hESCs). This transcript is characterized by a novel exon 1 conserved only in a sub-group of anthropoids. The corresponding N-truncated protein, also expressed in embryoid bodies (EBs), has the same apparent molecular weight as a recently identified highly functional dystrophin [30, 31]. Future studies of this new human embryonic 412 kDa dystrophin isoform will contribute to a better understanding of DMD physiopathology. In addition, we demonstrated using a phosphorodiamidate morpholino oligomer (PMO) targeted to skip the *DMD* exon 53 that this robust BMP4-inducible hiPSCs model, providing large amount of a long *DMD* transcript and the corresponding protein, can be an efficient tool to accelerate the development of DMD genetic therapeutic approaches.

## Methods

### Ethics, consent, and permissions

All healthy individuals and DMD patients had written an informed consent before the biopsy procedure.

At the Cochin Hospital-Cochin Institute, the collection of primary cultures of myoblasts was established from patient muscle biopsies conducted as part of medical diagnostic procedure of neuromuscular disorders. For each patient included in this study, signed informed consent was obtained to collect and study biological resources, and establish primary cultures of fibroblasts and myoblasts at the Hospital Cell Bank-Cochin Assistance Publique—Hôpitaux de Paris (APHP) . This collection of myoblasts was declared to legal and ethical authorities at the Ministry of Research (number of declaration, 701, n° of the modified declaration, 701–1) via the medical hosting institution, APHP, and to the “Commission Nationale de l’Informatique et des Libertés” (CNIL) (number of declaration, 1154515).

The BMD muscle biopsy was carried out in accordance with the ethical rules of the institutions involved under approvals of the Nationwide Children’s Hospital Institutional Review Board.

### Cell culture

Human adult myoblasts from healthy individuals and DMD patients were provided by Celogos and Cochin Hospital-Cochin Institute (Additional file 1: Table S1). In Celogos laboratory, cell preparation was done according to patent US2010/018873 A1.

Cells were maintained in a myoblast medium: DMEM/F-12, HEPES (31330–038, Thermo Fisher Scientific, MA, USA) supplemented with 10 % fetal bovine serum (FBS, Hyclone, Logan, UT), 10 U/mL penicillin/streptomycin (15140122, Thermo Fisher Scientific) on 0.1 % gelatin (G1393, Sigma-Aldrich®, St. Louis, MO, USA) coated cultureware with 10 ng/mL fibroblast growth factor 2 (FGF2) (100-18B, Peprotech, Rocky Hill, NY), and Dexamethasone 50 nM (D4902, Sigma-Aldrich®).

For hiPSCs generation, plasmid MSCV-IRES-GFP (pMIG) vectors containing complementary deoxyribonucleic acids (cDNAs) of v-myc avian myelocytomatosis viral oncogene homolog (C-MYC), POU class 5 homeobox 1 or OCT-4 (POU5F1), SRY (sex determining region Y)-box 2 (SOX2), and Kruppel-like factor 4 (KLF4) were kindly provided by George Q. Daley [32]. These plasmids were individually transfected using Lipofectamine® 2000 (11668–027, Thermo Fisher Scientific) into platinum (PLAT)-A packaging cells (for amphotropic retroviral production, RV-102, Cell Biolabs, San Diego, CA) in a biosafety level 3 laboratory or PLAT-E packaging cells (for ecotropic retroviral production, RV-101, Cell Biolabs) in a biosafety level 2 laboratory. PLAT cells medium was replaced 24 h post-transfection: Dulbecco’s modified Eagle medium (DMEM), high glucose, GlutaMAX™ (31965023), pyruvate sodium 1 mM (11360–039), β-mercaptoethanol 50 μM (31350–010) (Thermo Fisher Scientific), and 10 % FBS (Hyclone).

At the same time, myoblasts were thawed and seeded at 1 × 10^5^ cells/cm^2^ in a 6-well plate.

One day after seeding, myoblasts for ecotropic reprogramming were treated for 1 h with murine cationic amino acid transporter-1 (mCAT-1) gesicles 2.5 × 10^−2^ μg/μL final concentration [33], in 500 μL/well of fresh myoblast medium. Viral supernatants were collected 48 h post-transfection, filtered through a 0.45-μm filter (146561, Dutscher SA, Brumath, France), and mixed at a 1:1:1:1 ratio in a tube already containing Polybren (4 μg/mL final concentration, H9268, Sigma-Aldrich®) and Hepes (0.01 M final concentration, 15630056, Thermo Fisher Scientific). One milliliter of this mix was added to each well of mCAT-1 gesicles-treated myoblasts.

The medium was changed the following day. Four days post-transduction, myoblasts were passaged and seeded at three densities, 1.5 × 10^4^, 3 × 10^4^, and 6 × 10^4^ cells/cm^2^, on feeder layers of Zenith CF1 MEF (ZFVC-001, IVF Online, Toronto, Canada), mitomycin-C (M4287, Sigma-Aldrich®) treated. After 24 h, reprogrammed myoblasts were shifted in “hESCs medium”: Knockout™ DMEM (10829–018), 20 % KnockOut™ Serum Replacement (10828–028), 1X MEM Non-Essential Amino Acids Solution (11140–035), 50 μM β-mercaptoethanol (31350–010), 2 mM GlutaMAX™ Supplement (35050–038), and 10 U/mL penicillin/streptomycin (15140–122) (Thermo Fisher Scientific), 10 ng/mL FGF2 (Peprotech), supplemented with 0.5 mM valproic acid (P4543, Sigma-Aldrich®) during 10 days, with medium change every 2 days.

Human myoblasts for amphotropic reprogramming were transduced by the amphotropic retrovector mix for 24 h. They were then passaged and seeded in myoblast medium at 3.4 × 10^3^ cells/cm^2^ per well in 0.1 % gelatin-coated 6-well plates. The following day, the reprogrammed myoblasts were shifted to hESCs medium with valproic acid treatment during 10 days and medium change every 2 days.

The hiPSCs colonies were picked between day 15 and day 40 after transduction. They were subsequently expanded with manual passages as clumps and maintained on mitomycin-C treated mouse embryonic fibroblasts (MEFs) in hESCs medium. StemMACS™ Y27632 (130-103-922, Miltenyi biotech, Bologna, Italy) was used at 10 μM to increase the seeding efficiency of hiPSCs for the initial colony expansion after picking, for the first passage and at thawing.

Human iPSCs and ESCs (Additional file 1: Table S1) were harvested for banking in single cell with StemPro® Accutase® (A11105-01, Thermo Fisher Scientific) and froze in Cryostor® CS10 (210102, BioLife Solutions, Inc., Bothell, WA).

Before our experiments, all hiPSCs and hESCs were adapted and maintained with mTeSR™1 culture medium (05850, Stemcell Technologies, Vancouver, Canada) on Corning® Matrigel® Basement Membrane Matrix, lactose dehydrogenase elevating virus (LDEV)-Free-coated cultureware (354234, Corning Incorporated, NY, USA).

For adaptation of hiPSCs and hESCs to mTeSR™1 culture system (05850, Stemcell Technologies), cells were thawed on mitomycin-C treated MEFs in mTeSR™1 medium (05850, Stemcell Technologies) with 10 μM StemMACS™ Y27632. After 6 days, the adaptation consisted of three successive passages on Corning® Matrigel®-coated (354234) cultureware with several differentiation removal steps. The cells were first passaged with collagenase type IV (7909, Stemcell Technologies), then with dispase (1 U/mL, 7923, Stemcell Technologies) and finally in single cell with StemPro® Accutase® (A11105-01, Thermo Fisher Scientific) with an average of 5 days between each passage. At this step, cells must be mainly undifferentiated to be successfully adapted to mTeSR™1 culture system (05850, Stemcell Technologies) and to be banked. Cells were then seeded, passaged and thawed each time with 10 μM StemMACS™ Y27632.

**Fig. 1:**
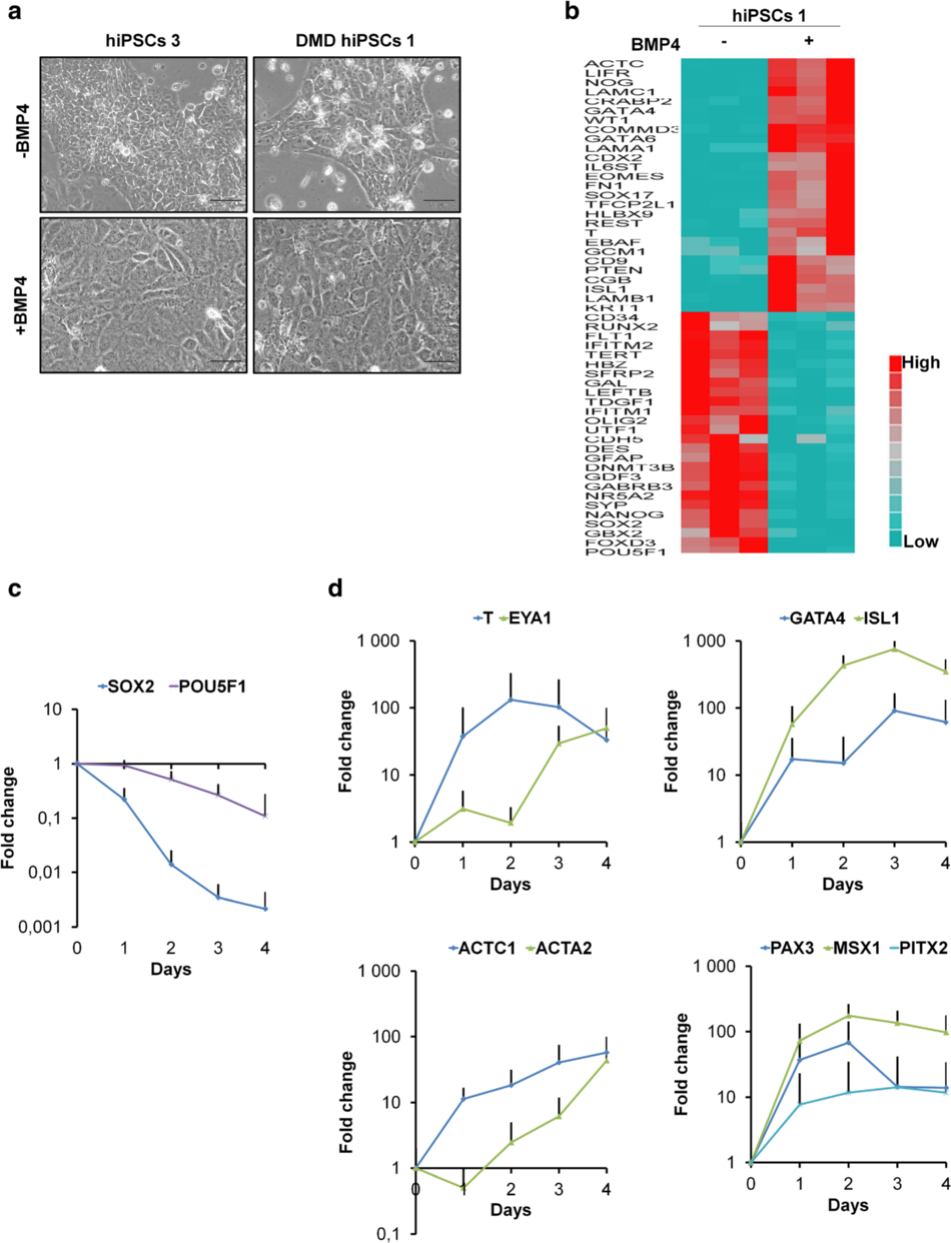
BMP4 treatment induces mesoderm lineage markers in hiPSCs/hESCs. **a** Examples of morphology in hiPSCs 3 and DMD hiPSCs 1 at day 4 either without or after a single BMP4 treatment. Scale bar = 50 μm. **b** TaqMan® Human Stem Cell Pluripotency Array data showing 2^-dCT^ of lineage marker genes in hiPSCs 1 at day 3 either without or after a single BMP4 treatment. Genes selected for display showed a minimum of ±2 fold change on relative quantifications (RQs) calculation in BMP4-treated hiPSCs 1, as compared to hiPSCs 1 without BMP4 treatment (*p* < 0.05; based on a Student’s *t* test). **c** and **d** Quantitative RT-PCR of **c** pluripotency and **d** early mesoderm specific genes. Curves represent the mean ± SD (standard deviation) from nine pluripotent stem cell lines (hPSCs) at days 0 through 4 after BMP4 treatment. Gene expression was normalized to the mean of glyceraldehyde-3-phosphate dehydrogenase (*GAPDH*) and ubiquitin C (*UBC*) and plotted (log10 scale) relative to the mean expression of all pluripotent stem cells at day 0 (D0)

We used for Fig. 1a the AnalySIS getIT 5.1 software with an Olympus CKX41 microscope and SC30 digital camera.

### Cell treatments

Pluripotent stem cells were thawed on Corning® Matrigel®-coated (354234) cultureware in mTeSR™1 (05850, Stemcell Technologies) with 10 μM StemMACS™ Y27632. When the cells were close to 70 % confluence, they were passaged and seeded at 4 × 10^4^ cells/cm^2^ with 10 μM StemMACS™ Y27632, with or without recombinant human BMP4 (314-BP-050, R&D Systems, Minneapolis, MN) at a final concentration of 5 ng/mL. Collections of RNAs or proteins were done without any further medium change. The medium was changed at day 4 only for collections between days 5 and 7.

Recombinant human noggin (NOG, 120-10C, Peprotech) was added for BMP4 inhibition at the seeding step at 100 ng/mL final concentration, 1 h prior BMP4 addition.

For Activin A treatment, after seeding of human pluripotent stem cells (hPSCs) as described above for BMP4 treatment, Activin A was added at 10 ng/mL (120–14; Peprotech).

### RNA purification and qRT-PCR

After harvesting pluripotent stem cells using StemPro® Accutase® (A11105-01, Thermo Fisher Scientific) and myoblasts/myotubes with Trypsin-ethylenediaminetetraacetic acid (EDTA) (25300–054, Thermo Fisher Scientific), cell pellets were resuspended in 350 μL of lysis buffer containing guanidine isothiocyanate (RLT) (79216, Qiagen, Hilden, Germany) and stored at −80 °C. Total RNA were extracted and purified according to either the RNeasy Mini Kit or Micro Kit protocol (74104 and 74004, Qiagen). Several human RNAs were used for the dystrophin protein 412 kDa embryonic (*Dp412e*) expression study (Testis 636533; Ovary 636555; Fetuses, 636185; Master Panel II, 636643; Cerebellum, 636535; Cerebral cortex 636561; Fetal heart, 636583; Smooth muscle, 636547; Heart, 636532; Skeletal muscle, 636534; Clontech, Palo Alto, CA). Muscle RNA from healthy individual biopsy was provided by Cochin Hospital-Cochin Institute.

RNA level and quality were checked using a Nanodrop spectrophotometer (ND-1000, Thermo Fisher Scientific Inc., USA). For each sample, 500 ng of total RNA were reverse transcribed with random primers (48190–011), oligo(dT) (SO131), and deoxynucleotide (dNTP) (10297–018) using Superscript® III reverse transcriptase (18080–044) (Thermo Fisher Scientific). Thermocycling conditions were 10 min, 25 °C; 60 min, 55 °C; and 15 min, 75 °C.

We amplified for quantitative real-time polymerase chain reaction (qRT-PCR) these cDNA using primers (Thermo Fisher Scientific) listed in Additional file 2: Table S2. They were designed using Primer 3 (http://primer3.ut.ee) [34], Primer blast (http://www.ncbi.nlm.nih.gov/tools/primer-blast) and amplifX (v1.5.4, by Nicolas Jullien; CNRS; Aix-Marseille University; http://crn2m.univ-mrs.fr/pub/amplifx-dist). The amplification efficiency of each primer set (except for QT primer and *Dp412e* forward primer) was preliminarily determined by running a standard curve. Detection was performed using a QuantStudio™ 12K Flex Real-Time PCR System (Thermo Fisher Scientific). Reactions were carried out in a 384-well plate, with 10 μL containing diluted cDNA and primers, as well as Luminaris Color HiGreen qPCR Master Mixes Low Rox (K0973, Thermo Fisher Scientific Inc.). Thermocycling conditions were 50 °C during 2 min, 95 °C during 10 min, followed by 45 cycles including 15 sec at 95 °C, 1 min at 60 °C plus a dissociation stage. All samples were measured in triplicate.

### Protein isolation and Western blot analyses

After three rinses with cold PBS 1X (w/o Ca^2+^ and Mg^2+^, D8537, Sigma-Aldrich®), protein extracts were isolated from cultured cells by scraping (010154, Dutscher) with an extraction protein buffer (75 mM Tris–HCl pH 6.8, 15 % sodium dodecyl sulfate (SDS), 5 % β-mercaptoethanol, 20 % glycerol, 4 × 10^−4^mg/μL bromophenol blue, Protease Inhibitor Cocktail diluted 1:100 (P8340, Sigma-Aldrich®), PhosSTOP tablet (04906845001, Roche Diagnostics Corp., Indianapolis, USA)). Protein extracts were then heated once 5 min at 95 °C. Depending on the viscosity of the protein extract, additional protein extraction buffer was needed for some samples before a second and sometimes a third step of 5-min heating. Protein Extracts were centrifuged 7 min at 16,000 g, and supernatants were kept at −80 °C. Muscle protein extracts from healthy individual biopsy were provided by Cochin Hospital-Cochin Institute. The Western blots were performed for dystrophin detection with Criterion™ XT Tris-Acetate Precast Gels 3–8 % (345-0129/30, Bio-Rad, Hercules, CA) and XT Tricine running buffer (161–0790, Bio-Rad). Samples were run at room temperature (RT) for 1 h and 15 min at 150 V (except for samples in Fig. 3c: 2 h and 30 min at 150 V with running buffer replenishing after 1 h and 15 min) with HiMark™ Pre-Stained Protein Standard (LC5699, Thermo Fisher Scientific). Gels were rinsed once in water and blotted for 11 min with “high molecular weight” program of TransBlot® Turbo™ transfer system (Bio-Rad) using Trans-Blot®Turbo™ Midi Nitrocellulose Transfer Packs (170–4159). Blots were blocked for 45 min with 5 % non-fat dried milk (170–6404, Bio-Rad) in phosphate-buffered saline tween (PBST) buffer (Phosphate-buffered saline 1X tablets, P4417, Sigma-Aldrich®; 0.1 % Tween® 20, 28829.296, VWR, West Chester, PA) followed by an overnight incubation at 4 °C under agitation with either NCL-DYS1 Dy4/6D3 (RRID: AB_442080, Novocastra Laboratories, Newcastle, UK) 1:30 or Manex6 [35] kindly provided by Prof. Glenn Morris (MDA Monoclonal Antibody Resource, Wolfson Centre for Inherited Neuromuscular Disease, Oswestry, UK) 1:30, Mandra1 clone 7A10 (exon 77; Developmental Studies Hybridoma Bank (DSHB), Iowa City, IA) 1:10, Manhinge4A clone 5C11 (exon 62; DSHB) 1:30, Manex7374A clone 10A11 (exon 73–74; DSHB) 1:30, Mandys19 clone 8F6 (exon 21; DSHB) 1:30, and Mandys101 clone 7D12 (exon 40–41; DSHB) 1:30, in 5 % non-fat dried milk in PBST buffer. After PBST rinses, horseradish peroxidase (HRP)-conjugated polyclonal rabbit anti-mouse (P0260, DAKO, Glostrup, Denmark) 1:5000 was used as a secondary antibody after DYS1 or ECL Anti-Mouse IgG, horseradish peroxidase-linked species-specific whole antibody from sheep 1:10,000 (NA931, GE Healthcare Life Sciences) after all the other primary dystrophin antibodies in 5 % non-fat dried milk in PBST, for 5 h at RT under agitation. The blots were rinsed with PBST before immuno-reactive bands were visualized using Amersham ECL Select Western Blotting Detection Reagent according to the manufacturer’s protocol (RPN2235, GE Healthcare Life Sciences, Buckinghamshire, UK) and the ImageQuant LAS 4000 mini system (GE Healthcare Life Sciences).

After DYS1 visualization, blots were rinsed in PBST and stripped with Restore™ Western Blot Stripping buffer (21059, Thermo Fisher Scientific) for 15 min at RT. After additional PBST rinses, blots were blocked again and incubated with mouse monoclonal anti-alpha Tubulin antibody (DM1A) (ab7291, Abcam, Cambridge, UK) 1:2500 in 5 % non-fat dried milk PBST, during 1 h at RT under agitation. After PBST rinses, ECL Anti-Mouse IgG, horseradish peroxidase-linked species-specific whole antibody from sheep 1:10,000 (NA931, GE Healthcare Life Sciences) was used as a secondary antibody in 5 % non-fat dried milk PBST, for 1 h at RT under agitation. Amersham ECL Prime Western Blotting Detection Reagent (RPN2232, GE Healthcare Life Sciences) was used for immuno-reactive bands visualization.

α-Tubulin detection was done either after DYS1 for the blots on Fig. 3, in Additional file 3: Figure S5a and Additional file 4: Figure S6b or in parallel to DYS1 and other dystrophin antibodies for all the other blots presented in this study by cutting the membranes before primary antibody incubation.

Western blots were performed for Phospho-small mothers against decapentaplegic (SMAD)1/5 detection with Criterion™ TGX™ Precast Gels 4–15 % (5671084, Bio-Rad) and Tris/Glycine/SDS running buffer (1610772, Bio-Rad). Samples were run at RT for 55 min at 200 V. Gels were rinsed once in water and blotted for 7 min with “mixed MW” program of TransBlot® Turbo™ transfer system (Bio-Rad).

Blots were blocked for 1 h with 5 % non-fat dried milk in tris-buffered saline tween (TBST) buffer at RT (137 mM NaCl, 20 mM Tris pH 7.6, 0.1 % Tween® 20 (28829.296, VWR)) followed by an overnight incubation at 4 °C under agitation with Phospho-SMAD1/5 antibody (Ser463/465, 41D10; 9516P, Cell Signaling, Beverly, MA) 1:1000 in 10 % bovine serum albumin (BSA) TBST. After TBST rinses, ECL Anti-Rabbit IgG, HRP-linked (NA934, GE Healthcare Life Sciences) 1:10,000 was used as a secondary antibody in 5 % non-fat dried milk TBST, for 1 h at RT under agitation. The blots were rinsed with TBST before immuno-reactive bands visualization with Amersham ECL Prime. α-Tubulin detection was then done after a stripping step (see above).

Quantification showed in Additional file 4: Figure S6c was done with the ImageQuant TL 7.0 software.

### 5′RACE PCR

Characterization of transcripts by rapid amplification of cDNA 5′ ends (5′RACE) polymerase chain reaction (PCR) was performed using the SMARTer® RACE cDNA Amplification Kit (634923, Clontech) according to the manufacturer instructions. RNA of the precursors derived from hiPSCs 1 3 days after BMP4 treatment was used for this purpose. Briefly, integrity of purified RNAs was checked upon migration in an agarose gel stained with Ethidium Bromide. RNA (500 ng) was used as a template to produce the RACE-ready cDNAs whose 5′ extremities are modified to incorporate the Universal Primer (UPA) sequence. A first round of cDNA amplification was performed using a first set of primers (Forward: UPA long: 5′-CTAATACGACTCACTATAGGGCAAGCAGTGGTATCAACGCAGAGT-3′, Reverse: DP427R1: 5′-TGTAGGTCACTGAAGAGGTTCTCAA-3′ corresponding to a region in the third exon of *DMD* cDNA). Products of amplification were next amplified by nested primers (Forward: NUPA: 5′-AAGCAGTGGTATCAACGCAGAGT-3′, Reverse: DP427R2: 5′-TGTGCATTTACCCATTTTGTG-3′ corresponding to a region in the second *DMD* exon). To increase the specificity of the RACE assay, priming of cDNAs synthesis was also achieved using a *DMD*-specific primer in the fourth exon (Ex4R1: 5′-GGGCATGAACTCTTGTGGAT-3′) instead of the polyT primer included in the SMARTer® RACE cDNA Amplification Kit. cDNAs obtained with this modified protocol were next amplified by primers Forward: UPA long: 5′-CTAATACGACTCACTATAGGGCAAGCAGTGGTATCAACGCAGAGT-3′, Reverse: 602Rex3: 5′-GGTCTAGGAGGCGCCTCCCATCCTGTAG-3′), followed by a nested PCR using Forward: NUPA: 5′-AAGCAGTGGTATCAACGCAGAGT-3′, Reverse: 603Rexnew: 5′-TCCACACCAGGTGGGGACGGATGACCT-3′ corresponding to *Dp412e* exon 1.

Amplicons from the nested PCRs were finally cloned using the Zero Blunt® PCR cloning kit (K2700-20, Thermo Fisher Scientific) prior sequencing (GATC Biotech, Konstanz, Germany). Overlapping reads were assembled into contigs mapped on the *DMD* gene.

### Immunolabeling

After 4 days with or without BMP4 treatment, slides for immunolabeling were prepared with the Thermo Scientific™ Cytospin™ 4 Cytocentrifuge. Briefly, cultures were washed once with PBS 1X (P4417, Sigma-Aldrich®), and StemPro® Accutase® (A11105-01, Thermo Fisher Scientific) was added for 5 min at 37 °C. The cells were harvested and transferred in a 15-mL tube, and the enzyme was diluted in DMEM/F-12, HEPES (31330–038, Thermo Fisher Scientific). Cells were counted and resuspended at 400,000-cells/mL concentration. Five hundred microliter of the cell suspension were loaded in each Shandon cytospin® centrifuge funnels, such as a spot of 200,000-cells was available on each superfrost® plus slide (631–0108, VWR) at the end of the centrifugation cycle (900 rpm, 4 min). Cells were immediately fixed with 4 % paraformaldehyde (15710, Euromedex, Souffelweyersheim, France) for 10 min at RT and then washed three times in PBS 1X for 15 min each.

Cell spots were delimited for immunolabeling with a PAP pen (Z672548-1EA, Sigma-Aldrich**®**) and then permeabilized with PBS 1X containing 0.3 % Triton™ X-100 (T8787, Sigma-Aldrich**®**) for 30 min at RT. Incubation with pure NCL-DYS1 Dy4/6D3 (Novocastra Laboratories) was performed overnight at 4 °C, then slides were washed three times with PBS 1X for 30 min each wash. Incubation with a mix of Dylight 549-conjugated AffiniPure F(ab′)2 fragment Goat Anti-Mouse IgG (H+L) (1:1000; 115-506-003, Jackson ImmunoResearch laboratories Inc., PA, USA) and DAPI (1:2000; 268298, Calbiochem, La Jolla, CA) was done for 30 min at RT. Slides were finally washed three times with PBS 1X (30 min/wash) and mounted with fluoromount (F4680; Sigma-Aldrich**®**). Observation and image captures were done on an AxioObserver Z1 Zeiss microscope with Zen Blue software (Carl Zeiss Microscopy GmbH, Jena, Germany).

For Fig. 3d, 40 % of brightness were added in Microsoft® PowerPoint® 2010 after the TIFF image insertion of hiPSCs 1 and DMD hiPSCs 2.

### PCR and TaqMan® arrays analyses

TaqMan® Array Human Stem Cell Pluripotency Panel (4385344, Thermo Fisher Scientific) was used to investigate the differentiation status of BMP4-treated hiPSCs 1 at day 3 as compared to untreated hiPSCs 1. According to TaqMan® Array protocol (Thermo Fisher Scientific), instead of 500 ng, we used 1500 ng of total RNA for each sample to do the reverse transcription (for details, see “RNA Purification and qRT-PCR” paragraph above). Each cDNA sample was mixed with 2X TaqMan® Gene Expression Master Mix (4369016, Thermo Fisher Scientific) and distributed in triplicates on the TaqMan® arrays. qRT-PCR were performed on an Applied Biosystems 7900HT Fast Real-Time PCR System (2 min, 50 °C; 10 min, 95 °C; 40X (15 s, 95 °C; 60 s, 60 °C)), and raw data were formatted to be analyzed using the online SAbioscience tool.

TGF-β/BMP signaling pathway study was performed using 384 wells RT^2^ Profiler Plus PCR Array (PAHS-035YE, Qiagen) according to the Qiagen Handbook. After total RNA isolation using RNeasy Mini kit (74104, Qiagen), 500 ng of RNA for each sample were treated with Buffer GE (Qiagen; 5 min, 42 °C) to eliminate genomic DNA. Purified RNAs were then reverse transcribed using the RT^2^ First Strand Kit (330401, Qiagen; 5X Buffer BC3, Control P2, RE3 Reverse Transcriptase Mix; 15 min, 42 °C then 5 min, 95 °C). Each cDNA sample was complemented with Qiagen 2X RT^2^ SYBR Green Mastermix (330521, Qiagen) and distributed in quadruplicates in the PCR arrays.

Three samples were tested at day 3: hiPSCs 1 without treatment, after BMP4, or NOG + BMP4 treatment. Analyses (ΔCt, ΔΔCt, Fold Change, statistical analyses) were performed using the online SAbioscience tool associated to the RT^2^ PCR arrays (http://pcrdataanalysis.sabiosciences.com/pcr/arrayanalysis.php).

### Embryoid bodies

We used the Stemcell Technologies protocol to produce embryoid bodies (EBs). Briefly, pluripotent stem cells were thawed on Corning® Matrigel®-coated (354234) flasks in mTeSR™1 (05850, Stemcell Technologies) with 10 μM StemMACS™ Y27632. When the cells were close to 70 % confluence, they were harvested using StemPro® Accutase® (A11105-01, Thermo Fisher Scientific) and resuspended in AggreWell™ medium (05893, StemCell Technologies) supplemented with 10 μM StemMACS™ Y27632 and finally seeded on AggreWell™ TM800 culture plates (27865, Stemcell Technologies). According to the StemCell procedure, three million cells were thus distributed on each well to form a maximum of 300 EBs with 10,000 cells per EB. EBs were maintained in Aggrewell plates during the whole culture duration to avoid EBs fusion and to preserve a homogenous differentiation. Half of the culture media was gently replaced every 2 days. For BMP4 treatment, BMP4 was added directly in Aggrewell™ medium at EBs seeding (30 ng/mL) and every medium change. To harvest EBs, the whole culture medium of a well was pipetted up and down, transferred to a 40 μm strainer (352340, Dutscher) to eliminate any single cell, and each well was washed twice with DMEM/F-12, HEPES (31330–038, Thermo Fisher Scientific). EBs were then transferred to a new tube with DMEM/F-12, HEPES, centrifuged 5 min at 300 g, resuspended in buffer RLT (79216, Qiagen) or protein extraction buffer (see “Protein isolation and Western Blot analyses” section), and stored at −80 °C for subsequent analyses.

### Exon skipping

Two days after induction, BMP4-treated hiPSCs 1 were transfected by electroporation with a phosphorodiamidate morpholino oligomer (PMO) targeting exon 53 of the *DMD* gene, based on previously published data [36] at 1, 10, or 100 μM with 200,000 cells in 20 μL solution from the P3 Primary Cell 4D-Nucleofector® X Kit (V4XP-3032, Lonza, Basel, Switzerland) using the CB150 program on the 4D-Nucleofector™ System (Lonza). RNA extraction was carried on transfected cells 24 h later followed by reverse transcription as described above. PCR was done on 250 ng cDNA using forward and reverse primers (Fw 5′-TTACCGACTGGCTTTCTCTGC-3′ and Rv 5′-GTCTGCCACTGGCGGAGGTC-3′, Thermo Fisher Scientific) and Taq DNA polymerase (10342, Thermo Fisher Scientific) as described by the manufacturer’s instructions, for a final reaction volume of 50 μL. PCR reaction started by a step at 94 °C for 3 min, followed by 35 cycles at 94 °C for 45 s, 55 °C for 45 s and 72 °C for 45 s, and a final step at 72 °C for 5 min. Exon skipping was analyzed using the DNA 1000 kit (5067, Agilent, Santa Clara, CA, USA) on the Agilent 2100 Bioanalyzer. Full-length PCR product was 460 bp and exon skipped length PCR product was 248 bp. Results are displayed as a gel-like image and computed by the Agilent 2100 Bioanalyzer software v3.81.

### Statistical analyses

Except for the PCR arrays, all statistical analyses were done with JMP® (v9.0.2). We used Wilcoxon’s test for qRT-PCR analyses. No statistical difference was found between DMD hiPSCs and normal hPSCs at day 0 and every day after BMP4 induction until day 4. Therefore, DMD patients and healthy individuals were analyzed together for this study. Exceptionally, BMP4-treated DMD hiPSCs 2 sample was removed from the analysis of the primer set specific to exons 20–21, as we did not detect any *DMD* expression due to the patient genetic mutation.

## Results

### BMP4-treated human pluripotent stem cells express mesoderm markers

BMP4 was used to induce differentiation toward mesodermic lineage of human pluripotent stem cells lines (hPSCs; including hiPSCs and hESCs; Additional file 1: Table S1).

When hPSCs without BMP4 treatment started to form compacted colonies, BMP4-treated hPSCs formed a confluent layer with a more flattened morphology (Fig. 1a).

TaqMan® Array showed, on BMP4-treated hiPSCs 1, the downregulation of pluripotency markers (e.g., *SOX2*, *POU5F1*) and upregulation of markers specific to mesoderm (e.g., *T*, *EOMES*, *CDX2*, ISL LIM homeobox 1 (*ISL1*), GATA binding protein 4 (*GATA4*), and actin, alpha, cardiac muscle 1 (*ACTC1*)) or to endoderm (such as *SOX17*) but none to ectoderm (such as paired box 6 (*PAX6*)) (Fig. 1b).

qRT-PCR, on all BMP4-treated hPSCs, confirmed the downregulation of pluripotency markers (high for *SOX2* and moderate for *POU5F1*; Fig. 1c) and upregulation of mesodermic markers (Fig. 1d) that had been previously identified (i.e., *T*, *ISL1*, *GATA4*, and *ACTC1*) or additionally tested (i.e., paired-like homeodomain transcription factor 2 (*PITX2*), Msh homeobox 1 (*MSX1*), *PAX3*, EYA transcriptional coactivator and phosphatase 1 (*EYA1*), actin, alpha 2, smooth muscle, aorta (*ACTA2*)).

### BMP4-treated human pluripotent stem cells express a long *DMD* transcript with a novel exon 1 conserved in a sub-group of anthropoids

**Fig. 2:**
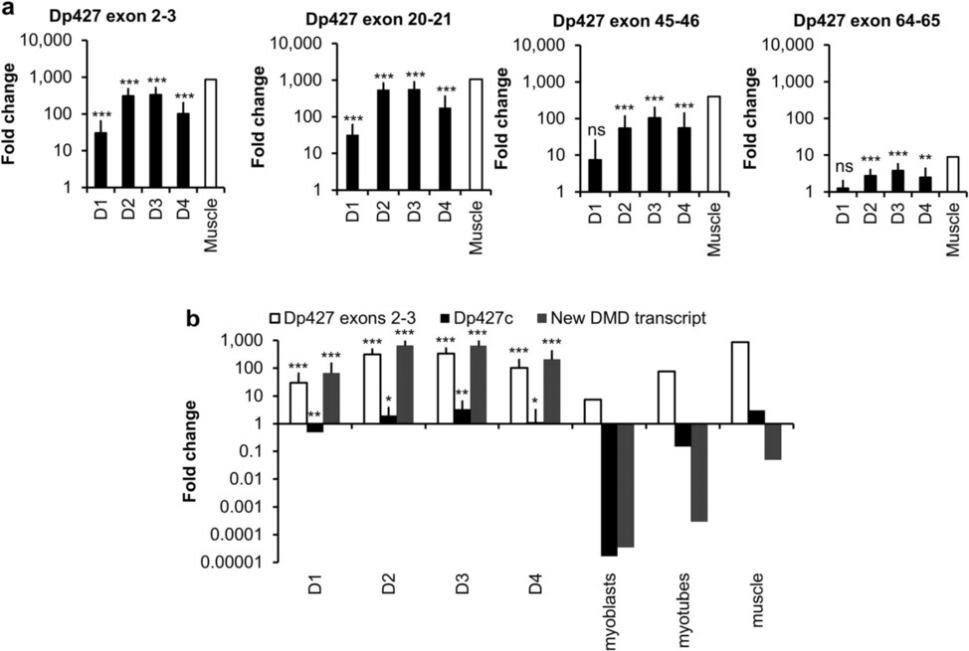
BMP4-treated hPSCs express a new transient long DMD transcript. **a** qRT-PCR of *DMD* transcripts using primers specific to exons 2–3, 20–21, 45–46, and 64–65. *Black bars* represent mean ± SD from nine hPSCs at days 0 through 4 after BMP4 treatment. A muscle biopsy from a healthy individual was used as a control (*white bar*). Gene expression was normalized to mean of *GAPDH* and *UBC* and plotted (log10 scale) relative to mean expression of all hPSCs at D0. **b** qRT-PCR of *DMD* transcripts using primers specific to exons 2–3, *Dp427c*, and the new *DMD* transcript. On the left side, data represent the mean ± SD from nine hPSCs from days 0 through 4 after BMP4 treatment. On the right are data from expression in cultured normal myoblasts and myotubes, as well as in muscle biopsied from a healthy individual. Gene expression was normalized to mean of *GAPDH* and *UBC* and plotted (log10 scale) relative to mean expression of all hPSCs at D0. *p < 0.05, **p < 0.01, ***p < 0.001, and ns for not statistically significant

After BMP4 treatment, all the primers designed along the *DMD* gene (Additional file 2: Table S2) demonstrated the robust expression of long *DMD* transcripts in all tested hPSCs, rising from day 1 to day 3, with high levels comparable to the levels of *Dp427m* expressed in normal adult skeletal muscle [37] (Fig. 2a). The difference of *DMD* transcript expression was statistically significant for all primers from day 2 in comparison to day 0 (exons 2–3, 310-fold, *p* < 0.0001; exons 20–21, 490-fold, *p* = 0.0002; exons 45–46, 54-fold, *p* < 0.0001; exons 64–65, 2.7-fold, *p* < 0.0001). Higher fold changes were always detected with the primers specific to the 5′-end (exons 2–3 and 20–21) compared to primers targeting the 3′-end (exons 45–46 and 64–65), as has already been described for *Dp427m* in normal muscles from mouse [38] and human [30, 39].

BMP4-treated hiPSCs 1 and DMD hiPSCs 3 were further analyzed to assess the *DMD* transcript expression over time. In both cell lines, the expression of *DMD* transcripts started to decrease from day 4 until day 7 and almost returned to basal level (hiPSCs 1: day 3 = 200-fold, day 7 = 7-fold; DMD hiPSCs 3: day 3 = 388-fold, day 7 = 6-fold; Additional file 5: Figure S1).

Specific primers for each known long *DMD* transcript were then designed to identify the BMP4-induced *DMD* transcripts. In BMP4-treated hPSCs, only a slight level of *Dp427c* was detected as in normal adult skeletal muscle (Fig. 2b). *Dp427c* expression was increased from day 1 and started to decrease at day 4 (day 3 vs day 0, 3.3-fold, *p* = 0.0035). However, *Dp427c* upregulation was too faint to explain the high level of *DMD* transcripts observed previously. We then decided to use 5′RACE PCR to identify a possible new transcript. Two main sequences were recursively found (Additional file 6: Figure S2). The first one was identified as the 164 last nucleotides of the Dp427c exon 1, spliced to the first nucleotides of exon 2. The second was a new sequence of 643 nucleotides (position in chromosome X (chrX), 33101211–33101853; (GenBank: KT072086)) spliced to the first nucleotides of exon 2. A corresponding transcript was not yet described, but a BLAST-like alignment tool (BLAT) analysis [40] showed that this sequence of 643 nucleotides was intronic and located 44.3 kb downstream of the dystrophin protein 427 kDa purkinje (Dp427p) exon1 and 62.9 kb upstream of exon 2.

With primers specific to this new exon 1, we thus found evidence that this new *DMD* transcript was expressed in all BMP4-treated hPSCs but neither in normal adult myoblasts/myotubes nor in normal adult skeletal muscle (Fig. 2b). The expression of this new *DMD* transcript rose from day 1 to day 3 after BMP4 treatment (day 3 vs day 0, 633-fold, *p* < 0.0001).

Genomic conservation analysis on 100 vertebrate species indicated that this new exon 1 sequence is only found in a sub-group of anthropoids despite some insertions and nucleotide substitutions between species (Additional file 7: Figure S3a). Furthermore, upstream and downstream of this new exon 1, there are sequences of 1.1 kb (chrX 33,101,854-33,103,030) and 6.3 kb (chrX 33,094,869-33,101,210), respectively, conserved only in these anthropoids despite gap regions in Marmoset, Gibbon, and Baboon. Interestingly, this large conserved sequence is composed of a long terminal repeat (LTR) retroelement split by inserts of simple repeats and Alu sequences (Additional file 7: Figure S3b). This LTR retroelement includes the whole human endogenous retrovirus-like sequence-Proline 1 (HuERS-P1) [41] flanked by two almost identical LTR8.

### The new long *DMD* transcript encodes an N-truncated dystrophin protein

Due to our previous analyses, we assumed that the new *DMD* transcript has the same sequence between exon 2 to 79 as the long known *DMD* transcripts. This theoretical sequence was analyzed to study the possible translated proteins (Additional file 8: Figure S4). No open reading frame appeared to be available from this new exon 1. The first start codon (AUG) corresponding to a methionine and allowing a long protein translation was identified in the sixth exon (chrX 32834745), followed closely thereafter by a second AUG in the same exon (chrX 32834733). If translation starts at this first or second AUG, the corresponding protein is predicted to be about 412 kDa with an N-terminal truncated actin-binding domain 1 (ABD1).

**Fig. 3:**
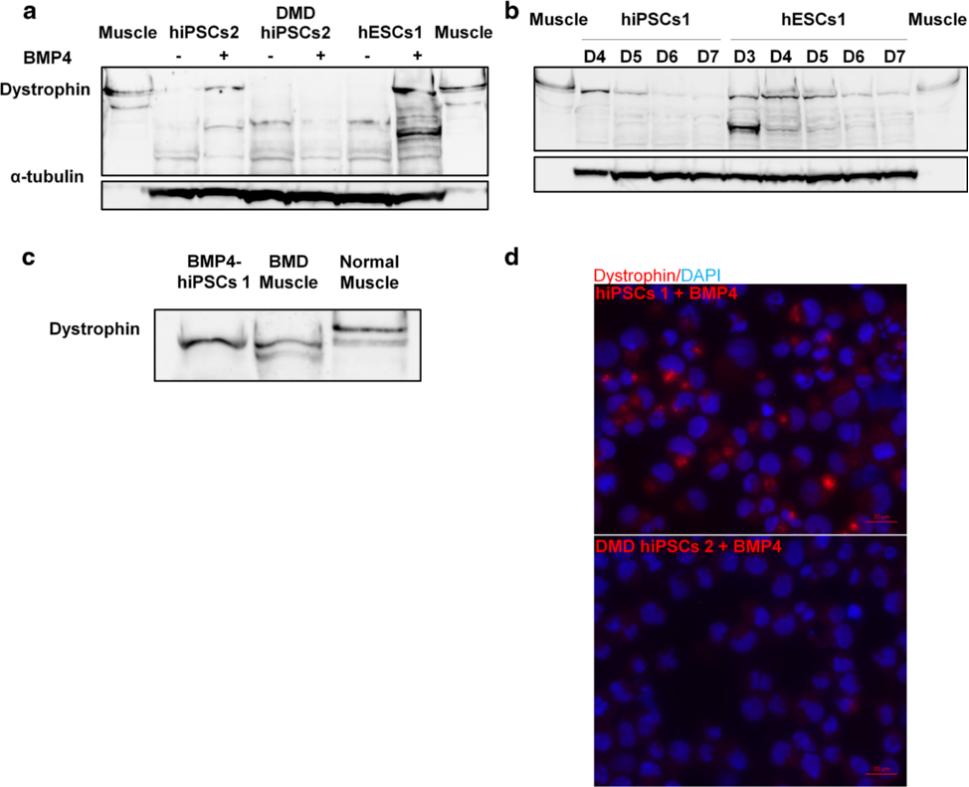
BMP4-treated hiPSCs/hESCs express dystrophin protein. **a** Western blot in three pluripotent stem cell lines (hiPSCs 2, DMD hiPSCs 2, and hESCs 1) at day 4 either without or after BMP4 treatment. **b** Western blot in hiPSCs 1 and hESCs 1 from days 3 through 7 following BMP4 treatment. **c** Western blot analysis on protein extracts from hiPSCs 1 4 days after BMP4 treatment, from a BMD patient muscle biopsy with an exon 2 frameshift mutation (c.40_41del (p.Glu14ArgfsX17)) and from normal skeletal muscle. **d** Immunolabeling of hiPSCs 1 and DMD hiPSCs 2, 4 days after BMP4 treatment. Scale bar = 20 μm. (Dystrophin antibody: DYS1 (RRID: AB_442080) for Western blots and immunolabeling; muscle biopsy protein extract from a healthy individual serves as a control; α-tubulin was used as loading control)

To investigate the presence of this protein, we decided to perform Western blot analyses 4 days after BMP4 treatment (Fig. 3a and Additional file 3: Figure S5a). In contrast to DMD hiPSCs, a protein just below 427 kDa was easily detected in all other BMP4-treated hPSCs.

A kinetic analysis showed that this protein was detectable as soon as day 2 after BMP4 treatment (Additional file 3: Figure S5b), and antibodies specific to several regions of the dystrophin protein confirmed the detection of a high molecular weight protein (Additional file 4: Figure S6a). Apart from Mandra1 antibody, they all demonstrated that this protein has a molecular weight slightly smaller than 427 kDa in comparison to the Dp427 long isoforms expressed in muscle tissue.

Western blot analyses were then done on normal BMP4-treated hPSCs to study the protein stability over time (Fig. 3b and see Additional file 4: Figure S6b and S6c). Apart from hESCs 1 and hiPSCs 2, at day 7 the dystrophin protein was barely detectable in the BMP4-treated hPSCs.

In skeletal muscle of mild BMD patients with a variety of mutations in the 5′ exons, a dystrophin protein with a translation starting also at exon 6 was recently described [30, 31]. Interestingly, one of these BMD patients, with an exon 2 frameshift mutation leading to an alternative translation initiation in exon 6 (Fig. 3c), has the same apparent molecular weight than our BMP4-induced dystrophin protein. Thus, the new dystrophin isoform described here is strongly suggested to be translated from exon 6 and therefore will be hereafter referred to as “Dp412e” (“e” standing for embryonic).

This dystrophin protein was also detected by immunofluorescence in BMP4-treated hiPSCs 1 but not in BMP4-treated DMD hiPSCs 2, confirming Western blot experiments (Fig. 3d). A large proportion of BMP4-treated hiPSCs 1, after 4 days of treatment, was positive for dystrophin staining.

### BMP pathway activation is essential for *Dp412e* expression

**Fig. 4:**
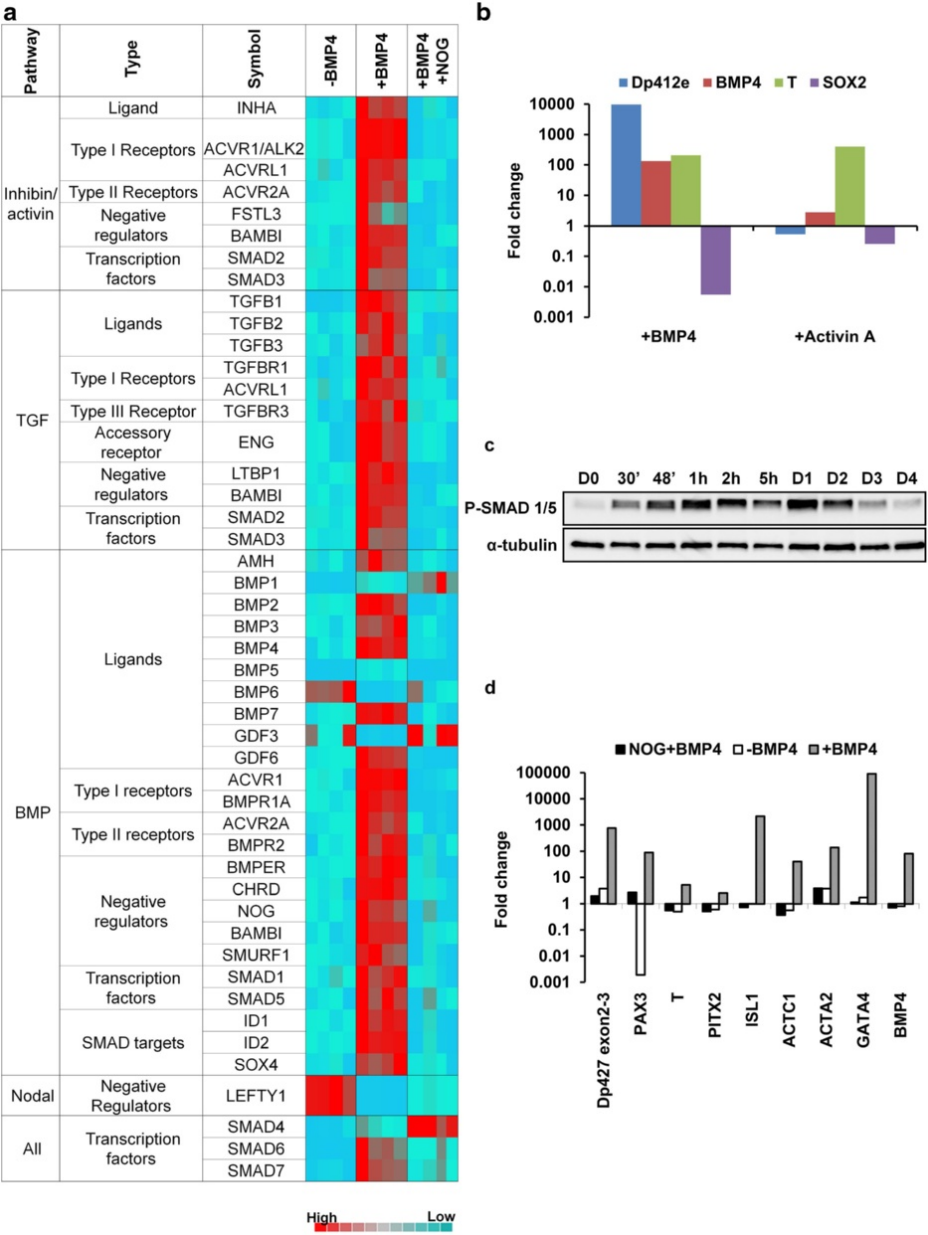
Correlation of BMP pathway activation with mesodermal commitment and Dp412e expression. **a** Microarray data showing 2^-dCT^ of Human TGF-β/BMP Signaling Pathway genes in hiPSCs 1 without or 3 days following a single BMP4 treatment or with addition of NOG prior to BMP4 treatment (four technical duplicates/condition). Genes selected for display showed a minimum of ±2-fold change on RQs calculation in either BMP4-treated hiPSCs 1 or NOG/BMP4-treated hiPSCs 1, as compared to hiPSCs 1 without BMP4 treatment (*p* < 0.05; based on a Student’s *t* test). **b** Quantitative RT-PCR in hiPSCs 1 of *Dp412e*, *BMP4*, *SOX2*, and *T* expression after 3 days of BMP4 or Activin A treatment. Gene expression was normalized to peptidylprolyl isomerase A or cyclophilin A (*PPIA*) and plotted (log10 scale) relative to the mean expression of hiPSCs 1 at day 3 without treatment (*n* = 2). **c** Western blot analysis of phospho-SMAD 1/5 in hiPSCs 1 from days 0 through 4 after BMP4 treatment. α-Tubulin was used as loading control. **d** Quantitative RT-PCR in hiPSCs 1 of *DMD* transcripts with specific primers to exons 2–3, *PAX3*, *T*, *PITX2*, *ISL1*, *ACTC1*, *ACTA2*, *GATA4*, and *BMP4* 3 days after BMP4 treatment, with or without Noggin (NOG) pre-treatment. Gene expression was normalized to *GAPDH* and plotted (log10 scale) relative to hiPSCs 1 at D0

PCR arrays were used to evaluate the impact of BMP4 treatment on signaling among the whole TGF-β superfamily, including the BMP pathway. They showed upregulation of BMP pathway actors, such as several ligands (*BMP2*, *4*, and *7*), type I and II receptors (*BMPR1A*, *ACVR1A*, and *BMPR2*, *ACVR2A*), *SMAD1* and *SMAD5*, *SMAD4* and inhibitors (*NOG*, *CHRD*, *BAMBI*, *SMAD6*, and *7*; Fig. 4a). Concerning the TGF-β pathway, type I receptor was upregulated but not type II. The Activin pathway actors were also upregulated with *SMAD2*, *SMAD3*, and the receptors cited above. However, 72 h following Activin A treatment, unlike *brachyury* (*T)*, a specific mesoderm marker, *Dp412e* was not induced by the Activin A pathway (Fig. 4b). Moreover, under this condition, the level of *SOX2* remained high while *endogenous BMP4* was not upregulated.

To confirm the BMP pathway activation, we investigated the phosphorylation of SMAD1 and SMAD5, two major proteins transducing extracellular signals. The phosphorylated SMAD1 and SMAD5 were detected as soon as 30 min after BMP4 addition, started to decrease at day 2, and returned to basal level at day 4 (Fig. 4c).

To explore the relationship between BMP4, mesoderm commitment and upregulation of *Dp412e*, noggin (NOG), a natural antagonist of BMP4, was used 1 h prior to BMP4 induction. In the presence of NOG, the BMP pathway actors mentioned above were not upregulated except *SMAD4* and *SMAD7* (Fig. 4a) and no *Dp412e* upregulation was detected (Fig. 4d). Other transcripts (Fig. 4d), previously shown to be upregulated by BMP4, were either slightly expressed (i.e., *PAX3*) or stayed at basal level (i.e., *T*, *PITX2*, *ISL1*, *ACTC1*, *ACTA2*, *GATA4*, and *BMP4*).

### Dp412e is expressed in embryoid bodies

To define more precisely the expression profile of *Dp412e*, RNAs from fetal/adult human tissues and human fetuses were analyzed. While expression of *Dp427m* and *Dp427c* was detected in expected tissues (Additional file 9: Figure S7), *Dp412e* was upregulated in BMP4-treated hiPSCs 1 but no significant expression was detected in all other tested RNAs from fetal and adult tissues.

**Fig. 5:**
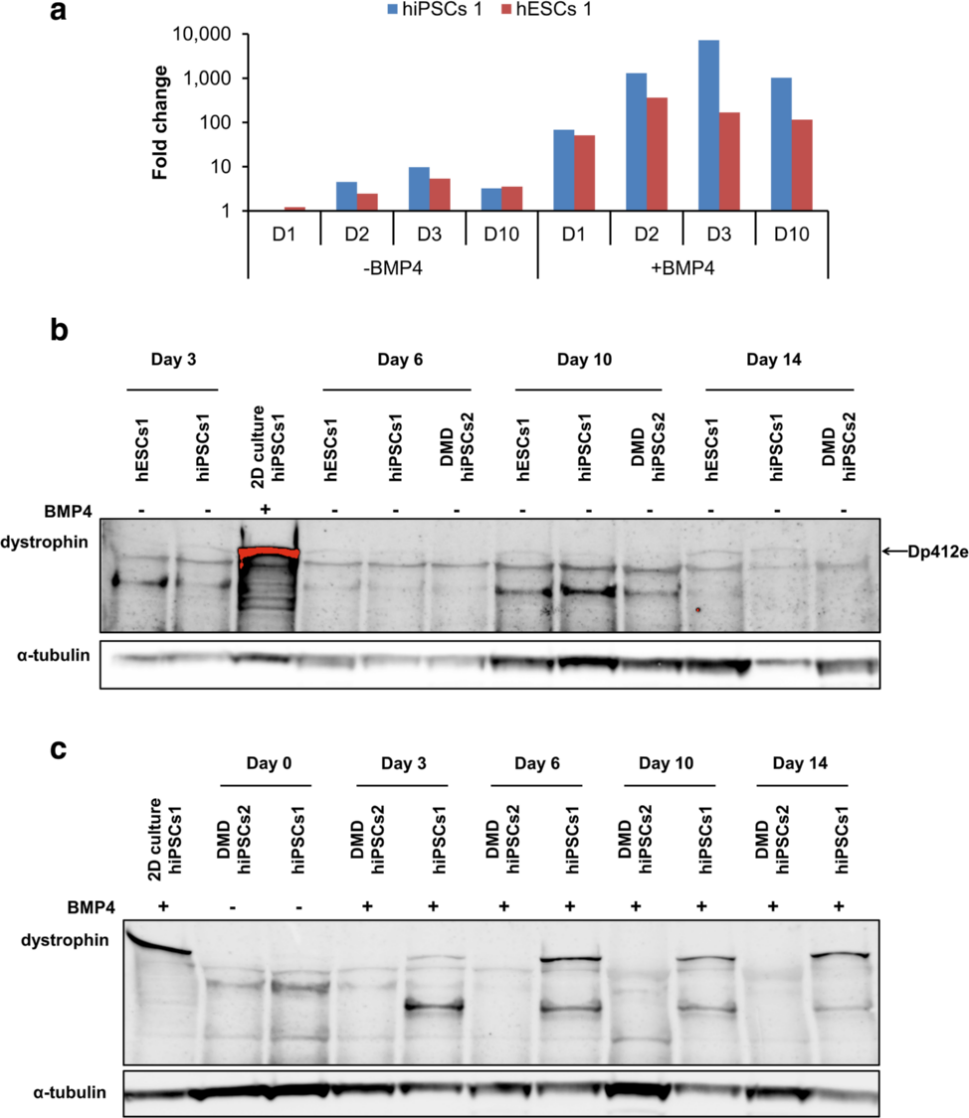
Dp412e is expressed in embryoid bodies (EBs). **a** Quantitative RT-PCR of *Dp412e* transcripts in hiPSCs 1 and hESCs 1 EBs from day 0 to day 10 of differentiation with or without BMP4 treatment. Gene expression was normalized to *PPIA* and plotted (log10 scale) relative to each cell line control at D0. **b** Western blot analysis on hESCs 1, hiPSCs 1, and DMD hiPSCs 2 EBs from day 3 to day 14 without BMP4 treatment. **c** Western blot analysis on hiPSCs 1 and DMD hiPSCs 2 EBs from day 0 to day 14 with BMP4 treatment. (Dystrophin antibody: DYS1; BMP4-treated hiPSCs 1 in 2 dimensions (2D) culture (4 days after treatment) was used as control; α-tubulin was used as loading control)

As seeking Dp412e expression in an exhaustive collection of human embryos remains a difficult task, we decided to study Dp412e profile in EBs. These aggregates of pluripotent stem cells mimic the early phases of development through differentiation toward the three germ layers [42]. *Dp412e* transcripts were detected at low level between day 2 and day 10 of differentiation in hESCs 1 and hiPSCs 1 EBs (Fig. 5a). With BMP4 treatment, *Dp412e* expression was strongly increased and was detected from day 1 at a level similar to BMP4-treated hPSCs (Fig. 5a). A faint band corresponding to Dp412e was detected by Western blot from day 3 to day 14 in hESCs 1 and hiPSCs 1 EBs without BMP4 treatment (Fig. 5b). As with *Dp412e* transcripts, a BMP4 treatment on hiPSCs 1 increased the Dp412e protein level during the EB differentiation from day 3 to day 14 (Fig. 5c). Moreover, Dp412e was not detected in DMD hiPSCs 2 EBs with or without BMP4 treatment (Fig. 5b, c).

### Exon skipping on BMP4-treated hiPSCs 1: a proof of concept

The BMP4-treated hPSCs model provides an unlimited quantity of cells expressing a large amount of *Dp412e* transcript and the corresponding protein only 72 h following BMP4 treatment. Furthermore, this fast, easy, and robust cell platform is already adapted to high-throughput and high-content screening approaches. As we can study the protein rescue in a timescale of only a week, this model could efficiently allow the validation and the improvement of DMD genetic therapeutic approaches.

The exon-skipping strategy was chosen to do the proof of concept, using a phosphorodiamidate morpholino oligomer (PMO) targeting exon 53 based on previously published data [36].

**Fig. 6:**
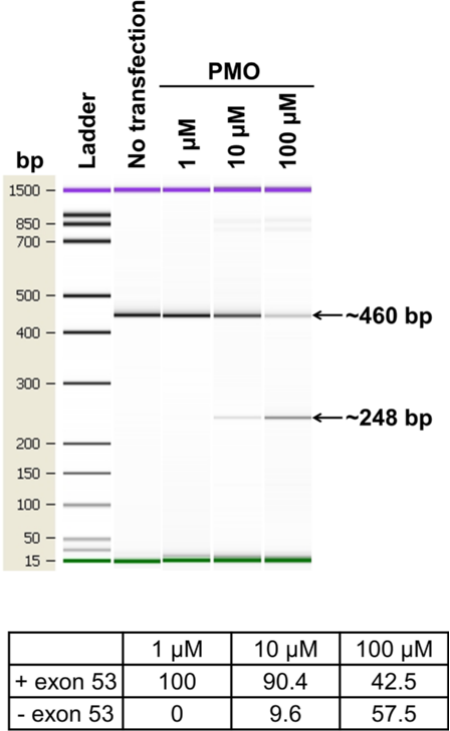
Exon skipping on BMP4-treated hiPSCs 1. Gel-like image data showing exon skipping results 3 days after BMP4 treatment and 24 h after phosphorodiamidate morpholino oligomer (PMO) transfection. Full-length PCR product is 460 bp and exon skipped length PCR product is 248 bp. The percentages of exon skipping obtained with the PMO exon 53 were calculated by the Agilent 2100 Bioanalyzer software

Three days after BMP4 treatment and 1 day after PMO transfection on hiPSCs 1, a dose-dependent skipping effect of the PMO 53 was observed. Indeed, no exon skipping was detectable with 1 μM PMO, whereas nearly 10 % of the total amount of *Dp412e* had a skipped exon 53 with 10 μM and almost 58 % with 100 μM (Fig. 6).

## Discussion

This study identifies a new long dystrophin isoform, herein denominated “Dp412e”, specific to early stages of differentiation induced by BMP4 in human pluripotent stem cells.

The specificity of this new *DMD* transcript is determined by a newly identified exon 1. *Dp412e* exon 1 and its associated upstream/downstream sequences are composed of simple repeats [43], Alu sequences [44, 45], and a LTR retroelement [46]. This LTR retroelement could be a relic of an ancient retrovirus infection over 40 million years ago in a common ancestor of the sub-group of anthropoids highlighted here. The *DMD* gene is known to have acquired new sequences and become more complex during evolution [6, 47]. This notably occurred at different stages of anthropoid evolution, as demonstrated for the *Dp412e* exon 1 region, *Dp427m* promoter with its upstream region, and for exon X and exon 2a which have been described in *DMD* transcripts [48–50]. Insertion in the human genome of Alu sequences participate in gene regulation [51], and retroelements can bring tissue-specific gene expression like for amylase in parotid [52, 53] or new functional proteins like syncytins in placenta [54, 55]. Retrovirus-like sequences can also provide functional modules including enhancers, promoters and splice sites [56], inducing the expression of new sequences. The exon X supports the idea that *Dp412e* exon 1 insertion in a *DMD* transcript could be the result of this phenomenon, as it is also located in the same retrovirus-like sequence HuERS-P1, 4.7 kb downstream of *Dp412e* exon 1.

Unlike the *Dp412e* exon 1 conservation in a sub-group of anthropoids, a similar embryonic dystrophin protein could be expressed in ancient species with a different exon 1 sequence due to strong positive selection inducing independent convergent evolution, as has been shown with salivary amylase in mice and human, and with syncytins in diverse mammalian species [52–54].

Moreover, our study indicates that the two first in-frame AUGs in exon 6 are the starting point of the Dp412e translation. The functionality of these AUGs has already been explored *in vitro* using reporter constructs [31]. Changing the first AUG to non-initiation codon moderately decreased the translation yield whereas changing the second AUG almost abrogated it. The *DMD* transcript secondary structure, predicted *in silico* (http://mfold.rna.albany.edu/?q=mfold/rna-folding-form), could form a hairpin loop that brings these two AUGs side by side; therefore, we can hypothesize that they are closely related.

With a translation starting at exon 6, it implies that the first five exons in the 5′-end are not translated.

To have one or several exons untranslated is not uncommon, as shown for amylase [53], prolactin receptor [57] and *Dp140* [58]. The 5′-untranslated region (5′-UTR) of *Dp412e* resembles that of the *Dp140* transcript, as the *Dp140* exon 1 is also located in an intron (the 44^th^) and its translation starts in exon 51 [58].

For 5′-UTRs, one of the possible functions is the internal ribosome entry site (IRES) allowing the translation to begin downstream of the first AUG codon in a cap-independent manner [59, 60]. A glucocorticoid-responsive IRES in exon 5 of the *DMD* gene has already been identified using reporter assays and confirmed in vivo in mice and *in vitro* using human muscular cells to induce translation initiation in exon 6 [30]. A detailed analysis of whether the exon 5 IRES is utilized in translation of *Dp412e* will require a significant number of additional experiments to address and is beyond the scope of this current study. However, we note that in our hands, neither dexamethasone (1 μM and 10 nM) nor methylprednisolone (3 μM and 10 nM) treatment succeeded in modifying Dp412e protein expression in BMP4-treated hiPSCs 1 (data not shown). As suggested in the original report [30], glucocorticoid action on this IRES could be dependent on a muscular context which would be irrelevant in our BMP4-treated model or the Dp412e translation yield could already be at a maximum. Even if the corresponding transcripts are different, Dp412e protein was shown to have the same apparent molecular weight as the dystrophin protein of a BMD patient. Interestingly, these truncated dystrophin proteins are expressed in two radically different contexts. However, the mild BMD phenotype associated with this truncated dystrophin [61] indicates that Dp412e could also be functional. Dp412e possesses several regions along its sequence allowing interactions with different partners [62, 63] and even with a truncated ABD1; Dp412e could still bind actin with ABD2 in the rod domain [64].

Dp412e was detected only in early differentiated cells, i.e., BMP4-treated hPSCs and EBs. In a sustainable manner, EBs express low level of Dp412e in the absence of exogenous BMP4. In this particular context, BMP4 could still be involved, as *endogenous BMP4* transcripts were expressed at a basal level before and during EB formation and further differentiation (data not shown). In line with this hypothesis, EBs treated with BMP4 quickly express a larger stable amount of Dp412e.

Activation of BMP pathway is required for *Dp412e* expression in early mesoderm precursor cells. Nevertheless, the activation of *brachyury* (*T*) is insufficient to trigger *Dp412e* expression, as demonstrated by Activin A experiments, possibly due to the associated low BMP4 endogenous level with high level of SOX2 in contrast with the BMP4 experiments. Cells expressing *Dp412e* could represent a particular type of mesoderm whose function remains to be clarified.

Different dystrophin transcripts and isoforms are expressed due to alternative promoters and alternative splicing during development and/or among different tissues in rat, mouse, human and other vertebrates [3, 5–7, 65, 66]. More specifically, in mice and human, the splicing pattern evolves between *in utero* development and the adult state in the skeletal muscle, heart and brain [3, 4, 66, 67]. These data support altogether the possible existence of unknown dystrophin isoforms such as Dp412e identified in this study.

Long dystrophin isoforms have already been demonstrated to be expressed in human non-muscle cells like in brain [68] and in melanocytes [69]. The dystrophin functions in non-muscle cells were shown to be related among others to adhesion and migratory capacity in melanocytes [69], to neuron migration during fetal life [23], and to modulation of synaptic function and plasticity [70]. Recently, dystrophin has also been proposed as a tumor suppressor through the regulation of invasion and migration in myogenic cancers [71]. The definition of Dp412e function(s) at early developmental stages, so far not addressed, could allow a better understanding of DMD pathophysiology. As a first step, Dp412e partners can be identified by immunoprecipitation and mass spectrophotometry studies. Furthermore, it will be interesting to verify the *Dp412e* expression in embryos from human and others anthropoids. Until now, the earlier detection of dystrophin in human embryos was described at 4 weeks of gestation by immunofluorescence [72]. However, the nature of the dystrophin isoform(s) detected in this study remains to be elucidated.

Finally, using PMO to induce exon skipping, we validated BMP4-treated hPSCs as a model. Current cell models are limited and cannot easily validate in parallel the restoration of a disrupted reading frame and the synthesis of shortened BMD-like dystrophin protein [73, 74]. Interestingly, the simple, fast, and robust BMP4-inducible model highlighted here, providing large amount of a long *DMD* transcript and the corresponding protein, is already well-adapted to high-throughput and high-content screening approaches.

## Conclusions

This study validates the use of hiPSCs to analyze early phases of human development in normal and pathological contexts and has led to the discovery of a new human embryonic 412 kDa dystrophin isoform. Deciphering the regulation process(es) and the function(s) associated to this new isoform will contribute to a better understanding of the DMD physiopathology and potential developmental defects. Furthermore, the validation and improvement of DMD genetic therapeutic approaches, like exon skipping, gene editing and gene addition [10], can be accelerated with the powerful BMP4-inducible cell platform presented here.

## Additional files

Additional file 1: Table S1. Human embryonic and induced pluripotent stem cell line list. Details concerning the stem cell lines used in the present study. Additional file 2: Table S2. Primer list. Primers used in the present study. Additional file 3: Figure S5. BMP4-treated hiPSCs/hESCs express dystrophin protein. (a) Western blot in six pluripotent stem cell lines (hiPSCs 1, 3 and 4, DMD hiPSCs 1 and 3, hESCs 2) at day 4 either without or after BMP4 treatment. (b) Western blot in hiPSCs 1 from days 0 through 4 after a single BMP4 treatment. (Dystrophin antibody: DYS1; Muscle biopsy protein extract from a healthy individual serves as a control, α-tubulin was used as loading control). Additional file 4: Figure S6. Dystrophin protein expression. (a) Western blot analyses of protein extracts from hiPSCs 1 at day 4 either without or after BMP4 treatment using antibodies directed against different regions of dystrophin. (Dystrophin antibodies: Manex6 (exon 6); Mandys19 (exon 21), Mandys101 (exon 40–41), Manhinge4A (exon 62), Manex7374A (exon 73–74), Mandra1 (exon 77). (b) Western blot with DYS1 antibody in three pluripotent stem cell lines (hiPSCs 2 and 4, hESCs 2) from days 3 through 7 following BMP4 treatment. (c) Quantification of dystrophin protein levels from the Western blots (b) in Fig. 3 and (b) in Fig. S6 (Muscle biopsy protein extract from a healthy individual serves as a control and α-tubulin was used as loading control). Additional file 5: Figure S1. BMP4-treated hPSCs express a new transient long DMD transcript. qRT-PCR of *DMD* transcripts using primers specific to exons 2–3 in hiPSCs 1 and DMD hiPSCs 3 at days 3 through 7 after BMP4 treatment. For each cell line, gene expression was normalized to *GAPDH* and plotted (log10 scale) relative to the expression at D0. Additional file 6: Figure S2. 5′RACE reveals the presence of a novel exon 1. (a, b) Two sequences found several times by 5′RACE PCR on RNA from hiPSCs 1 three days after BMP4 treatment. In black are represented the nucleotides found to be spliced to the *DMD* gene exon 2 by 5′RACE PCR, with in (a), the 164 last nucleotides of *Dp427c* exon 1 and in (b), the new exon 1. In red are the parts of the RACE sequence corresponding to *DMD* gene exon 2. The black box in *Dp427c* sequence points out a nucleotide that does not match with Dp427c exon 1 reference sequence. The BLAT analyses were done on the web site https://genome.ucsc.edu. Additional file 7: Figure S3. The new DMD exon 1 belongs to a retrovirus-like sequence. (a) Alignment of the new exon 1 region (highlighted in light blue) among 100 vertebrate species with the conserved upstream/downstream sequence marked by red arrows (https://genome.ucsc.edu). (b) Schematic representation of the approximately 8 kb region in the *DMD* gene that is conserved among a sub-group of anthropoids. It is composed of simple repeats, Alu sequences and the whole human endogenous retrovirus-like sequence HuERS-P1 (http://www.dfam.org/entry/DF0000214) flanked by two LTR8 elements. Additional file 8: Figure S4.
*In silico* translation of the novel DMD transcript. Representation of the open reading frame (ORF) resulting in translation of the novel *DMD* transcript. Possible translated proteins from this ORF are represented in red. The largest predicted protein is composed of 3562 amino acids (≈412 kDa) (http://web.expasy.org/translate). Additional file 9: Figure S7. Dp412e expression study. Fold change obtained by quantitative RT-PCR using primers specific for *Dp427m*, *Dp427c* and *Dp412e* on RNAs from either human adult tissues, 7–11 weeks old fetuses or human 25–40 weeks old fetal tissues. Gene expression was normalized to *UBC* and relative to BMP4-treated hiPSCs 1 at day 3.

## Abbreviations

2D: 2 dimensions
5′RACE: rapid amplification of cDNA 5′ ends
ABD1: actin-binding domain 1
ACTA2: actin, alpha 2, smooth muscle, aorta
ACTC1: actin, alpha, cardiac muscle 1
BLAT: BLAST-like alignment tool
BMD: Becker muscular dystrophy
BMP4: bone morphogenetic protein 4
BSA: bovine serum albumin
c.40_41del: on cDNA, deletion of the 40th and 41st nucleotides from “A” of the first AUG
cDNA: complementary deoxyribonucleic acid
chrX: chromosome X
C-MYC: v-myc avian myelocytomatosis viral oncogene homolog
D0: day 0
dCt: delta threshold cycle
DMD: Duchenne muscular dystrophy
DMEM: Dulbecco’s modified Eagle medium
DNMT3B: DNA (cytosine-5-)-methyltransferase 3 beta
dNTP: deoxynucleotide
Dp412e: dystrophin protein 412 kDa embryonic
Dp427c: dystrophin protein 427 kDa cerebral
Dp427m: dystrophin protein 427 kDa muscular
Dp427p: dystrophin protein 427 kDa purkinje
DSHB: developmental studies hybridoma bank
EBs: embryoid bodies
EDTA: ethylenediaminetetraacetic acid
EYA1: EYA transcriptional coactivator and phosphatase 1
FBS: fetal bovine serum
FGF2: fibroblast growth factor 2
GAPDH: glyceraldehyde-3-phosphate dehydrogenase
GATA4: GATA binding protein 4
GRCh37/hg19: genome reference consortium human 37/human genome 19
hESCs: human embryonic stem cells
hiPSCs: human induced pluripotent stem cells
hPSCs: human pluripotent stem cells
HRP: horseradish peroxidase
HuERS-P1: human endogenous retrovirus sequence proline 1
IRES: internal ribosome entry site
ISL1: ISL LIM homeobox 1
kb: kilo bases
kDa: kiloDalton
KLF4: Kruppel-like factor 4
KSR: knockout serum replacement
LDEV: lactose dehydrogenase elevating virus
LTR: long terminal repeat
mCAT-1: murine cationic amino acid transporter-1
mdx: X-linked muscular dystrophy
MEF: mouse embryonic fibroblast
MSX1: Msh homeobox 1
NKX2.5: NK2 homeobox 5
NOG: noggin
ns: not statistically significant
OligodT: oligo deoxythymine
p.Glu14ArgfsX17: On the protein, the 14th amino acid (glutamic acid) is replaced by an arginine. It is a frameshift mutation with a stop codon 17 amino acids after this arginine
PAX: paired box
PBST: phosphate-buffered saline tween
PCR: polymerase chain reaction
PITX2: paired-like homeodomain transcription factor 2
PLAT: platinum
pMIG: plasmid MSCV-IRES-GFP
PMO: phosphorodiamidate morpholino oligomer
POU5F1: POU class 5 homeobox 1 or OCT-4
PPIA: peptidylprolyl isomerase A or cyclophilin A
qRT-PCR: quantitative real-time polymerase chain reaction
RLT: lysis buffer containing guanidine isothiocyanate
RQ: relative quantification
RT: room temperature
SD: standard deviation
SDS: sodium dodecyl sulfate
SMAD: small mothers against decapentaplegic
SOX: SRY (sex determining region Y)-box
T: Brachyury
TBST: tris-buffered saline tween
TGF-β: transforming growth factor beta
TGX: tris-Glycine eXtended
UBC: ubiquitin C
UCSC: University of California, Santa Cruz
UTR: untranslated region

## References

[1] Blake DJ, Weir A, Newey SE, Davies KE (2002). Function and genetics of dystrophin and dystrophin-related proteins in muscle. Physiol Rev.

[2] Muntoni F, Torelli S, Ferlini A (2003). Dystrophin and mutations: one gene, several proteins, multiple phenotypes. Lancet Neurol.

[3] Bies RD, Phelps SF, Cortez MD, Roberts R, Caskey CT, Chamberlain JS (1992). Human and murine dystrophin mRNA transcripts are differentially expressed during skeletal muscle, heart, and brain development. Nucleic Acids Res.

[4] Clerk A, Strong PN, Sewry CA (1992). Characterisation of dystrophin during development of human skeletal muscle. Development.

[5] Feener CA, Koenig M, Kunkel LM (1989). Alternative splicing of human dystrophin mRNA generates isoforms at the carboxy terminus. Nature.

[6] Jin H, Tan S, Hermanowski J, Böhm S, Pacheco S, McCauley JM (2007). The dystrotelin, dystrophin and dystrobrevin superfamily: new paralogues and old isoforms. BMC Genomics.

[7] Surono A, Takeshima Y, Wibawa T, Pramono ZA, Matsuo M (1997). Six novel transcripts that remove a huge intron ranging from 250 to 800 kb are produced by alternative splicing of the 5′ region of the dystrophin gene in human skeletal muscle. Biochem Biophys Res Commun.

[8] Darras BT, Miller DT, Urion DK, Pagon RA, Adam MP, Ardinger HH, Bird TD, Dolan CR, Fong C-T, Smith RJ, Stephens K (1993). Dystrophinopathies. GeneReviews(®).

[9] Flanigan KM (2014). Duchenne and Becker muscular dystrophies. Neurol Clin.

[10] Foster H, Popplewell L, Dickson G (2012). Genetic therapeutic approaches for Duchenne muscular dystrophy. Hum Gene Ther.

[11] Konieczny P, Swiderski K, Chamberlain JS (2013). Gene and cell-mediated therapies for muscular dystrophy. Muscle Nerve.

[12] Govoni A, Magri F, Brajkovic S, Zanetta C, Faravelli I, Corti S (2013). Ongoing therapeutic trials and outcome measures for Duchenne muscular dystrophy. Cell Mol Life Sci.

[13] Ruegg UT (2013). Pharmacological prospects in the treatment of Duchenne muscular dystrophy. Curr Opin Neurol.

[14] Guiraud S, Aartsma-Rus A, Vieira NM, Davies KE, van Ommen G-JB, Kunkel LM (2015). The pathogenesis and therapy of muscular dystrophies. Annu Rev Genomics Hum Genet.

[15] Tyler KL (2003). Origins and early descriptions of “Duchenne muscular dystrophy”. Muscle Nerve.

[16] Merrick D, Stadler LKJ, Larner D, Smith J (2009). Muscular dystrophy begins early in embryonic development deriving from stem cell loss and disrupted skeletal muscle formation. Dis Model Mech.

[17] Bassett DI, Bryson-Richardson RJ, Daggett DF, Gautier P, Keenan DG, Currie PD (2003). Dystrophin is required for the formation of stable muscle attachments in the zebrafish embryo. Development.

[18] Nguyen F, Cherel Y, Guigand L, Goubault-Leroux I, Wyers M (2002). Muscle lesions associated with dystrophin deficiency in neonatal golden retriever puppies. J Comp Pathol.

[19] Emery AEH (1977). Muscle histology and creatine kinase levels in the foetus in Duchenne muscular dystrophy. Nature.

[20] Toop J, Emery AE (1974). Muscle histology in fetuses at risk for Duchenne muscular dystrophy. Clin Genet.

[21] Vassilopoulos D, Emery AE (1977). Muscle nuclear changes in fetuses at risk for Duchenne muscular dystrophy. J Med Genet.

[22] Moat SJ, Bradley DM, Salmon R, Clarke A, Hartley L (2013). Newborn bloodspot screening for Duchenne muscular dystrophy: 21 years experience in Wales (UK). Eur J Hum Genet.

[23] Rosman NP, Kakulas BA (1966). Mental deficiency associated with muscular dystrophy. A neuropathological study. Brain.

[24] Pescatori M, Broccolini A, Minetti C, Bertini E, Bruno C, D’amico A (2007). Gene expression profiling in the early phases of DMD: a constant molecular signature characterizes DMD muscle from early postnatal life throughout disease progression. FASEB J.

[25] Takahashi K, Tanabe K, Ohnuki M, Narita M, Ichisaka T, Tomoda K (2007). Induction of pluripotent stem cells from adult human fibroblasts by defined factors. Cell.

[26] Bernardo AS, Faial T, Gardner L, Niakan KK, Ortmann D, Senner CE (2011). BRACHYURY and CDX2 mediate BMP-induced differentiation of human and mouse pluripotent stem cells into embryonic and extraembryonic lineages. Cell Stem Cell.

[27] Dosch R, Gawantka V, Delius H, Blumenstock C, Niehrs C (1997). Bmp-4 acts as a morphogen in dorsoventral mesoderm patterning in Xenopus. Development.

[28] Winnier G, Blessing M, Labosky PA, Hogan BL (1995). Bone morphogenetic protein-4 is required for mesoderm formation and patterning in the mouse. Genes Dev.

[29] Zhang P, Li J, Tan Z, Wang C, Liu T, Chen L (2008). Short-term BMP-4 treatment initiates mesoderm induction in human embryonic stem cells. Blood.

[30] Wein N, Vulin A, Falzarano MS, Szigyarto CA-K, Maiti B, Findlay A (2014). Translation from a DMD exon 5 IRES results in a functional dystrophin isoform that attenuates dystrophinopathy in humans and mice. Nat Med.

[31] Gurvich OL, Maiti B, Weiss RB, Aggarwal G, Howard MT, Flanigan KM (2009). DMD exon 1 truncating point mutations: amelioration of phenotype by alternative translation initiation in exon 6. Hum Mutat.

[32] Park I-H, Zhao R, West JA, Yabuuchi A, Huo H, Ince TA (2008). Reprogramming of human somatic cells to pluripotency with defined factors. Nature.

[33] Mangeot P-E, Dollet S, Girard M, Ciancia C, Joly S, Peschanski M (2011). Protein transfer into human cells by VSV-G-induced nanovesicles. Mol Ther.

[34] Untergasser A, Cutcutache I, Koressaar T, Ye J, Faircloth BC, Remm M (2012). Primer3—new capabilities and interfaces. Nucleic Acids Res.

[35] Bartlett RJ, Stockinger S, Denis MM, Bartlett WT, Inverardi L, Le TT (2000). In vivo targeted repair of a point mutation in the canine dystrophin gene by a chimeric RNA/DNA oligonucleotide. Nat Biotechnol.

[36] Popplewell LJ, Adkin C, Arechavala-Gomeza V, Aartsma-Rus A, de Winter CL, Wilton SD (2010). Comparative analysis of antisense oligonucleotide sequences targeting exon 53 of the human DMD gene: implications for future clinical trials. Neuromuscul Disord.

[37] Bovolenta M, Scotton C, Falzarano MS, Gualandi F, Ferlini A (2012). Rapid, comprehensive analysis of the dystrophin transcript by a custom micro-fluidic exome array. Hum Mutat.

[38] Spitali P, van den Bergen JC, Verhaart IEC, Wokke B, Janson AAM, van den Eijnde R (2013). DMD transcript imbalance determines dystrophin levels. FASEB J.

[39] Tennyson CN, Shi Q, Worton RG (1996). Stability of the human dystrophin transcript in muscle. Nucleic Acids Res.

[40] Kent WJ (2002). BLAT—the BLAST-like alignment tool. Genome Res.

[41] Harada F, Tsukada N, Kato N (1987). Isolation of three kinds of human endogenous retrovirus-like sequences using tRNA(Pro) as a probe. Nucleic Acids Res.

[42] Conley BJ, Young JC, Trounson AO, Mollard R (2004). Derivation, propagation and differentiation of human embryonic stem cells. Int J Biochem Cell Biol.

[43] Richards RI, Sutherland GR (1994). Simple repeat DNA is not replicated simply. Nat Genet.

[44] Deininger PL, Batzer MA (1999). Alu repeats and human disease. Mol Genet Metab.

[45] Cordaux R, Batzer MA (2009). The impact of retrotransposons on human genome evolution. Nat Rev Genet.

[46] de Parseval N, Heidmann T (2005). Human endogenous retroviruses: from infectious elements to human genes. Cytogenet Genome Res.

[47] Böhm SV, Roberts RG (2009). Expression of members of the dystrophin, dystrobrevin, and dystrotelin superfamily. Crit Rev Eukaryot Gene Expr.

[48] Fracasso C, Patarnello T (1998). Evolution of the dystrophin muscular promoter and 5′ flanking region in primates. J Mol Evol.

[49] Dwi Pramono ZA, Takeshima Y, Surono A, Ishida T, Matsuo M (2000). A novel cryptic exon in intron 2 of the human dystrophin gene evolved from an intron by acquiring consensus sequences for splicing at different stages of anthropoid evolution. Biochem Biophys Res Commun.

[50] Roberts RG, Bentley DR, Bobrow M (1993). Infidelity in the structure of ectopic transcripts: a novel exon in lymphocyte dystrophin transcripts. Hum Mutat.

[51] Britten RJ (1996). DNA sequence insertion and evolutionary variation in gene regulation. Proc Natl Acad Sci U S A.

[52] Ting CN, Rosenberg MP, Snow CM, Samuelson LC, Meisler MH (1992). Endogenous retroviral sequences are required for tissue-specific expression of a human salivary amylase gene. Genes Dev.

[53] Meisler MH, Ting CN (1993). The remarkable evolutionary history of the human amylase genes. Crit Rev Oral Biol Med.

[54] Lavialle C, Cornelis G, Dupressoir A, Esnault C, Heidmann O, Vernochet C (2013). Paleovirology of “syncytins”, retroviral env genes exapted for a role in placentation. Philos Trans R Soc Lond B Biol Sci.

[55] Lokossou AG, Toudic C, Barbeau B (2014). Implication of human endogenous retrovirus envelope proteins in placental functions. Viruses.

[56] Blikstad V, Benachenhou F, Sperber GO, Blomberg J (2008). Evolution of human endogenous retroviral sequences: a conceptual account. Cell Mol Life Sci.

[57] Hu Z-Z, Zhuang L, Meng J, Tsai-Morris C-H, Dufau ML (2002). Complex 5′ genomic structure of the human prolactin receptor: multiple alternative exons 1 and promoter utilization. Endocrinology.

[58] Lidov HG, Selig S, Kunkel LM (1995). Dp140: a novel 140 kDa CNS transcript from the dystrophin locus. Hum Mol Genet.

[59] Pesole G, Grillo G, Larizza A, Liuni S (2000). The untranslated regions of eukaryotic mRNAs: structure, function, evolution and bioinformatic tools for their analysis. Brief Bioinformatics.

[60] Pichon X, Wilson LA, Stoneley M, Bastide A, King HA, Somers J (2012). RNA binding protein/RNA element interactions and the control of translation. Curr Protein Pept Sci.

[61] Flanigan KM, Dunn DM, von Niederhausern A, Howard MT, Mendell J, Connolly A (2009). DMD Trp3X nonsense mutation associated with a founder effect in North American families with mild Becker muscular dystrophy. Neuromuscul Disord.

[62] Lai Y, Thomas GD, Yue Y, Yang HT, Li D, Long C (2009). Dystrophins carrying spectrin-like repeats 16 and 17 anchor nNOS to the sarcolemma and enhance exercise performance in a mouse model of muscular dystrophy. J Clin Invest.

[63] Roberts RG (2001). Dystrophins and dystrobrevins. Genome Biol.

[64] Amann KJ, Guo AW, Ervasti JM (1999). Utrophin lacks the rod domain actin binding activity of dystrophin. J Biol Chem.

[65] Nicholson LV, Davison K, Falkous G, Harwood C, O’Donnell E, Slater CR (1989). Dystrophin in skeletal muscle. I. Western blot analysis using a monoclonal antibody. J Neurol Sci.

[66] Rau F, Lainé J, Ramanoudjame L, Ferry A, Arandel L, Delalande O (2015). Abnormal splicing switch of DMD’s penultimate exon compromises muscle fibre maintenance in myotonic dystrophy. Nat Commun.

[67] Dickson G, Pizzey JA, Elsom VE, Love D, Davies KE, Walsh FS (1988). Distinct dystrophin mRNA species are expressed in embryonic and adult mouse skeletal muscle. FEBS Lett.

[68] Sogos V, Reali C, Fanni V, Curto M, Gremo F (2003). Dystrophin antisense oligonucleotides decrease expression of nNOS in human neurons. Brain Res Mol Brain Res.

[69] Pellegrini C, Zulian A, Gualandi F, Manzati E, Merlini L, Michelini ME (2013). Melanocytes—a novel tool to study mitochondrial dysfunction in Duchenne muscular dystrophy. J Cell Physiol.

[70] Haenggi T, Fritschy J-M (2006). Role of dystrophin and utrophin for assembly and function of the dystrophin glycoprotein complex in non-muscle tissue. Cell Mol Life Sci.

[71] Wang Y, Marino-Enriquez A, Bennett RR, Zhu M, Shen Y, Eilers G (2014). Dystrophin is a tumor suppressor in human cancers with myogenic programs. Nat Genet.

[72] Durand M, Suel L, Barbet JP, Beckmann JS, Fougerousse F (2002). Sequential expression of genes involved in muscular dystrophies during human development. Morphologie.

[73] O’Leary DA, Sharif O, Anderson P, Tu B, Welch G, Zhou Y (2009). Identification of small molecule and genetic modulators of AON-induced dystrophin exon skipping by high-throughput screening. PLoS One.

[74] Shoji E, Sakurai H, Nishino T, Nakahata T, Heike T, Awaya T (2015). Early pathogenesis of Duchenne muscular dystrophy modelled in patient-derived human induced pluripotent stem cells. Sci Rep.

