# Supplementary material for: Dp412e: a novel human embryonic dystrophin isoform induced by BMP4 in early differentiated cells
Source: Skelet Muscle. 2015 Nov 14;5:40. doi: 10.1186/s13395-015-0062-6 (PMC4644319; doi:10.1186/s13395-015-0062-6)
Supplement: Additional file 1: Table S1. — Human embryonic and induced pluripotent stem cell line list. Details concerning the stem cell lines used in the present study. [file 13395_2015_62_MOESM1_ESM.pdf]

**Alias**	**Cell Lines**	**Reprogramming method**	**Reprogrammed cells**	**Provided by**	***DMD* gene mutations**
**hESCs 1**	SA001	-	-	Cellartis AB	-
**hESCs 2**	H9	-	-	Wicell research institute	-
**hiPSCs 1**	VAX1024	Amphotropic retroviruses	Myoblasts	Celogos	-
**hiPSCs 2**	M00180	Ecotropic retroviruses	Myoblasts	Cochin Hospital-Cochin Institute	-
**hiPSCs 3**	M00194	Ecotropic retroviruses	Myoblasts	Cochin Hospital-Cochin Institute	-
**hiPSCs 4**	M00398	Ecotropic retroviruses	Myoblasts	Cochin Hospital-Cochin Institute	-
**DMD hiPSCs 1**	M00197	Ecotropic retroviruses	Myoblasts	Cochin Hospital-Cochin Institute	In-frame Duplication exons 3-26
**DMD hiPSCs 2**	M00202	Ecotropic retroviruses	Myoblasts	Cochin Hospital-Cochin Institute	Out-of-frame Deletion exons 8-43
**DMD hiPSCs 3**	M00418	Ecotropic retroviruses	Myoblasts	Cochin Hospital-Cochin Institute	Stop exon 7 c
