# Supplementary material for: Dp412e: a novel human embryonic dystrophin isoform induced by BMP4 in early differentiated cells
Source: Skelet Muscle. 2015 Nov 14;5:40. doi: 10.1186/s13395-015-0062-6 (PMC4644319; doi:10.1186/s13395-015-0062-6)
Supplement: Additional file 2: Table S2. — Primer list. Primers used in the present study. [file 13395_2015_62_MOESM2_ESM.pdf]

Targets	Primers	Amplicons (bp)
ACTC1 (NM_005159)	QT00205296[1]	105
ACTA2 (NM_001613.2, NM_001141945.1)	Fw 5'-TCCTCCCTTGAGAAGAGTTACGA-3'	445
	Rv 5'-GGAGCAGGAAAGTGTTTTAGAAGC-3'	
BMP4 (NM_130851.2, NM_130850.2, NM_001202.3)	Fw 5'-ATGATTCCTGGTAACCGAATGC-3'	93
	Rv 5'-CCCCGTCTCAGGTATCAAACT-3'	
DMD exons 2_3	Fw 5'-TCACAAAATGGGTAAATGCACAA-3'	73
(NM_004010.3,NM_004009.3,NM_004006.2,NM_000109.3)	Rv 5'-TGTAGGTCACTGAAGAGGTTCTCAA-3'	
DMD exons 20_21 (NM_004010.3, NM_004009.3, NM_004006.2,	Fw 5'-ACCCACCACCCCATCAGA-3'	69
NM_000109.3)	Rv 5'-CGGTTGACTTCATCCTTACAAATTT-3'	
DMD exons 45-46 (NM_004020.3, NM_004023.2, NM_004022.2, NM_004021.2, NM_004013.2, NM_004012.3, NM_004011.3,	Fw 5'-GGCAGGAGGTCTGCAAACAG-3'	145
NM_004010.3, NM_004009.3, NM_004006.2, NM_000109.3)	Rv 5'-GGTTCAAGTGGGATACTAGCAATGT-3'	
DMD exons 64-65 (NM_004020.3, NM_004023.2, NM_004022.2, NM_004021.2, NM_004018.2, NM_004017.2, NM_004016.2, NM_004015.2, NM_004014.2, NM_004013.2, NM_004012.3, NM_004011.3, NM_004010.3, NM_004009.3, NM_004006.2,	Fw 5'-GGCCCTTTGCTTGGATCTCT-3'	58
NM_000109.3, NM_004019.2)	Rv 5'-GTGCTGGTCCAAGGCATCA-3'	
DMD Dp427m (NM_004006.2)	Fw 5'-GTGGGAAGAAGTAGAGGACTGT-3'	129
	Rv 5'-TCCTGTAGGTCACTGAAGAGGT-3'	
DMD Dp427c (NM_000109.3)	Fw 5'-TGGCATGATGGAGTGACAGGA-3'	150
	Rv 5'-CCCATCCTGTAGGTCACTGAA-3'	
DMD Dp427p1p2 (NM_004009.3, NM_004010.3)	Fw 5'-GAAATGTCTGAGGTCTCATCTGATGA-3'	97
	Rv 5'-GCTGCTTCCCAAACTTAGAAAATTGTGC-3'	
Dp412e (used with the reverse primer of *DMD* exons 2_3 set)	Fw 5'-CCCAGCAATAAGGATGAAAGAGA-3'	116
EYA1 (NM_000503, NM_172058, NM_172059)	QT00070980[1]	110
GAPDH	QT00079247[1]	
GATA4 (NM_002052.3)	Fw 5'-CGACACCCCAATCTCGATATG-3'	
	Rv 5'-GTTGCACAGATAGTGACCCGT-3'	
ISL1 (NM_002202.2)	Fw 5'-CACACAGCGGAAACACTCGAT-3'	102
	Rv 5'-TACGGGATCAAATGCGCCAA-3'	
MSX1 (NM_002448)	QT00224350[1]	96
MYF5 (NM_005593.2)	Fw 5'-AACCCTCAAGAGGTGTACCAC-3'	208
	Rv 5'-AGGACTGTTACATTCGGGCAT-3'	
MYF6 (NM_002469.2)	Fw 5'-GGAGCGCCATCAGCTATATTG-3'	131
	Rv 5'-ATCCGCACCCTCAAGATTTTC-3'	
MYOD1 (NM_002478.4)	Fw 5'-GGGGCTAGGTTCAGCTTTCT-3'	129
	Rv 5'-CTACATTTGGGACCGGAGTG-3'	
MYOG (NM_002479.5)	Fw 5'-TAAGGTGTGTAAGAGGAAGTCG-3'	437
	Rv 5'-CCACAGACACATCTTCCACTGT-3'	
PAX3 (NM_181459.3, NM_001127366.2, NM_000438.5, NM_181458.3, NM_181457.3, NM_013942.4, NM_181460.3,	Fw 5'-AGTTCCATCAGCCGCATC-3'	95
NM_181461.3)	Rv 5'-TTCTTCTCGCTTTCCTCTGC-3'	
PAX7 (NM_002584.2, NM_013945.2, NM_001135254.1)	Fw 5'-CAAGATTCTTTGCCGCTACC-3'	384-390
	Rv 5'-TTCAGTGGGAGGTCAGGTTC-3'	
PITX2 (NM_153427.2,NM_153426.2,NM_001204399.1,NM_001204398.1,	Fw 5'-GCCGAGGACCCGTCTAAGA-3'	60
NM_001204397.1,NM_000325.5)	Rv 5'-CTGGCTGGTAAAGTGAGTCCG-3'	
POU5F1 endogenous[2] (NM_001285987.1,NM_203289.5,NM_001285986.1,NM_001173531.2,	Fw 5'-CCTCACTTCACTGCACTGTA-3'	164
NM_002701.5)	Rv 5'-CAGGTTTTCTTTCCCTAGCT-3'	
PPIA (NM_001300981.1, NM_021130.4)	Fw 5'-GGTGACTTCACACGCCATAATG-3’	103
	Rv 5'-ACAAGATGCCAGGACCCGTAT-3’	
SOX2 endogenous (NM_003106.3)	Fw 5'-CCGGTACGCTCAAAAAGAAA-3'	59
	Rv 5'-TGTCATTTGCTGTGGGTGAT-3'	
T (NM_003181.3,NM_003181.3)	Fw 5'-ATGAAGGCTCCCGTCTCC-3'	177
	Rv 5'-CACCGCTATGAACTGGGTCT-3'	
UBC (NM_021009.6)	Fw 5'-ATTTGGGTCGCGGTTCTTG-3'	133
	Rv 5'-TGCCTTGACATTCTCGATGGT-3'	

[1] Quantitect primer assay (Qiagen) [2] Park et al, 2008
