# Supplementary figures and images for: Dp412e: a novel human embryonic dystrophin isoform induced by BMP4 in early differentiated cells
Source: Skelet Muscle. 2015 Nov 14;5:40. doi: 10.1186/s13395-015-0062-6 (PMC4644319; doi:10.1186/s13395-015-0062-6)

**a**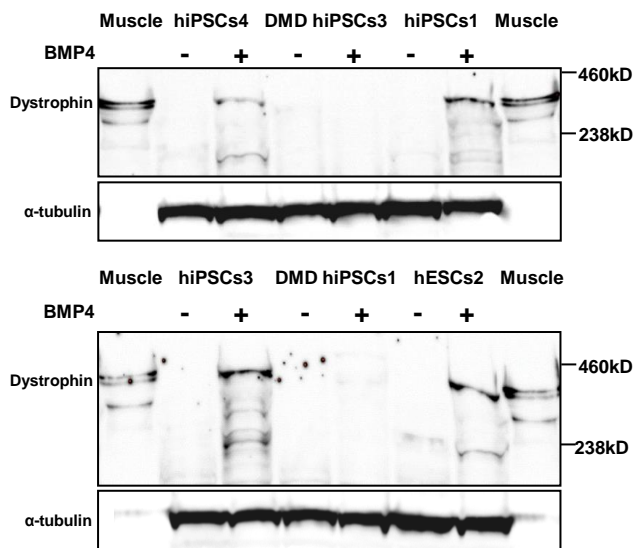**b**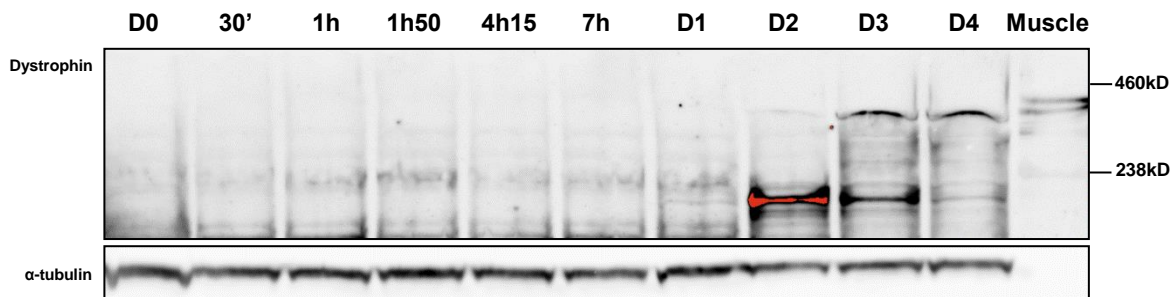

Supplement: Additional file 3: Figure S5. — BMP4-treated hiPSCs/hESCs express dystrophin protein. (a) Western blot in six pluripotent stem cell lines (hiPSCs 1, 3 and 4, DMD hiPSCs 1 and 3, hESCs 2) at day 4 either without or after BMP4 treatment. (b) Western blot in hiPSCs 1 from days 0 through 4 after a single BMP4 treatment. (Dystrophin antibody: DYS1; Muscle biopsy protein extract from a healthy individual serves as a control, α-tubulin was used as loading control). [file 13395_2015_62_MOESM3_ESM.pdf]

**a**

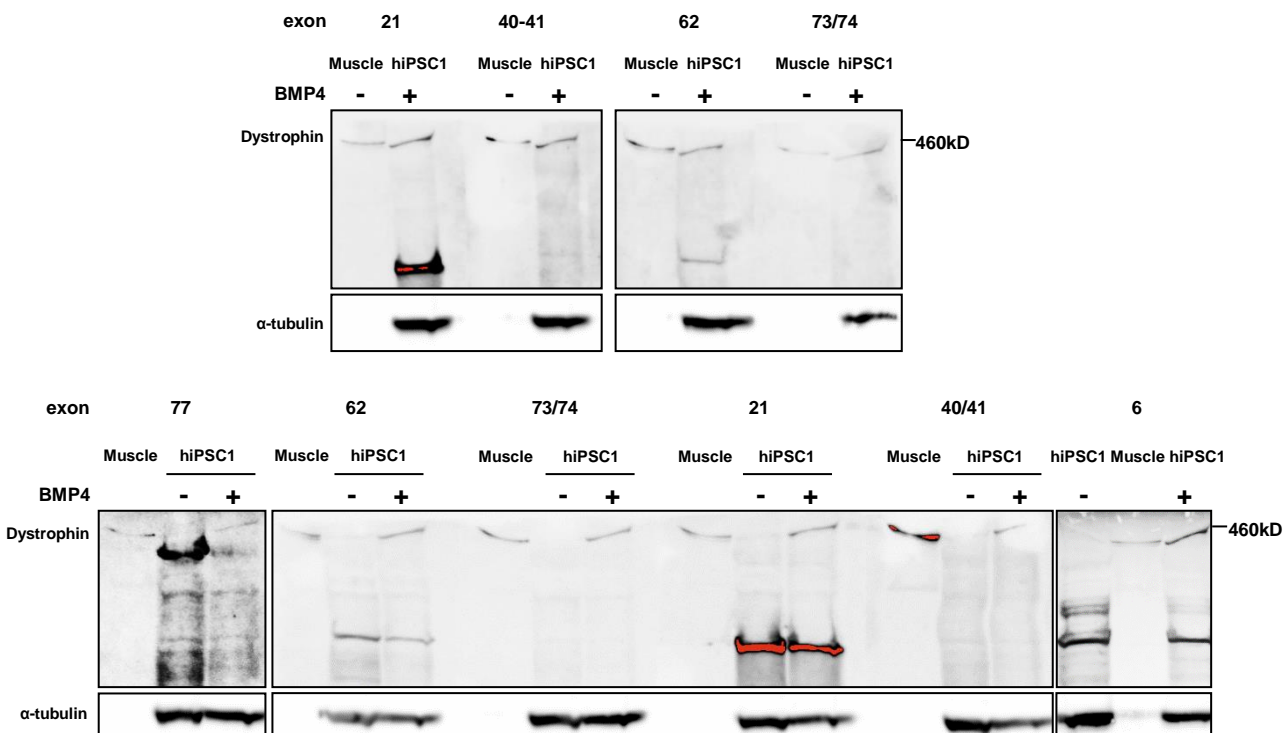

**b**

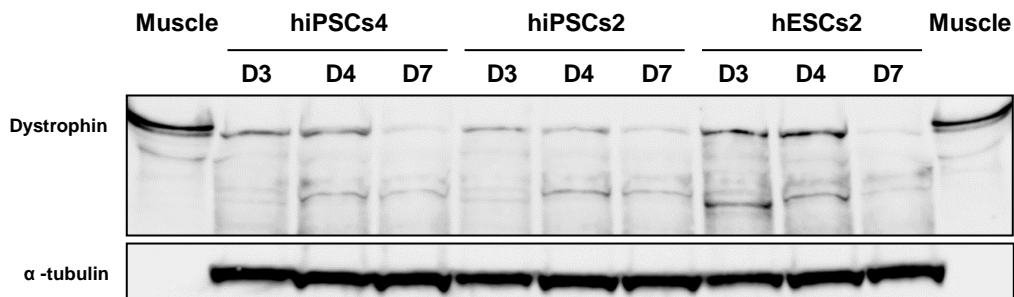

**c**

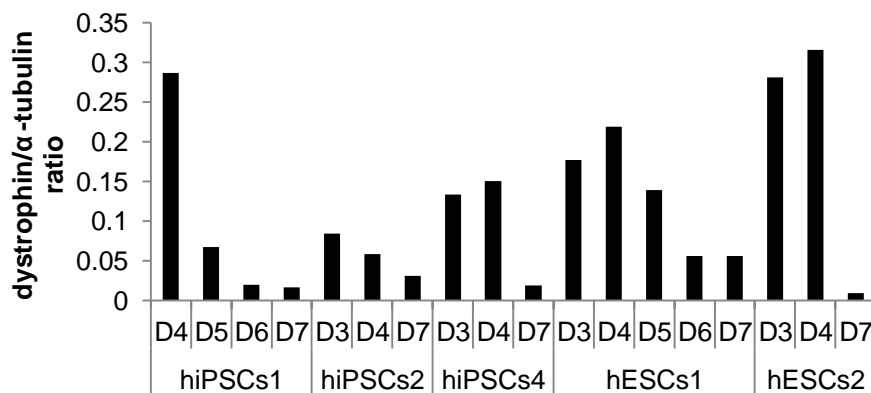

Supplement: Additional file 4: Figure S6. — Dystrophin protein expression. (a) Western blot analyses of protein extracts from hiPSCs 1 at day 4 either without or after BMP4 treatment using antibodies directed against different regions of dystrophin. (Dystrophin antibodies: Manex6 (exon 6); Mandys19 (exon 21), Mandys101 (exon 40–41), Manhinge4A (exon 62), Manex7374A (exon 73–74), Mandra1 (exon 77). (b) Western blot with DYS1 antibody in three pluripotent stem cell lines (hiPSCs 2 and 4, hESCs 2) from days 3 through 7 following BMP4 treatment. (c) Quantification of dystrophin protein levels from the Western blots (b) in Fig. 3 and (b) in Fig. S6 (Muscle biopsy protein extract from a healthy individual serves as a control and α-tubulin was used as loading control). [file 13395_2015_62_MOESM4_ESM.pdf]

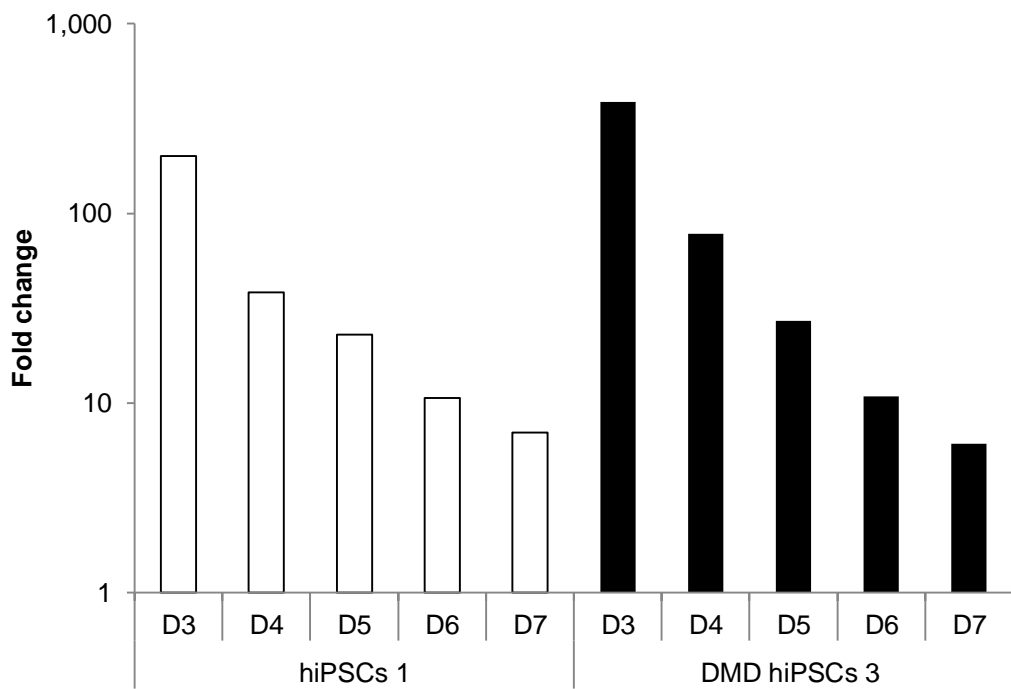

Supplement: Additional file 5: Figure S1. — BMP4-treated hPSCs express a new transient long DMD transcript. qRT-PCR of DMD transcripts using primers specific to exons 2–3 in hiPSCs 1 and DMD hiPSCs 3 at days 3 through 7 after BMP4 treatment. For each cell line, gene expression was normalized to GAPDH and plotted (log10 scale) relative to the expression at D0. [file 13395_2015_62_MOESM5_ESM.pdf]

a

5' Upstream

3' Downstream

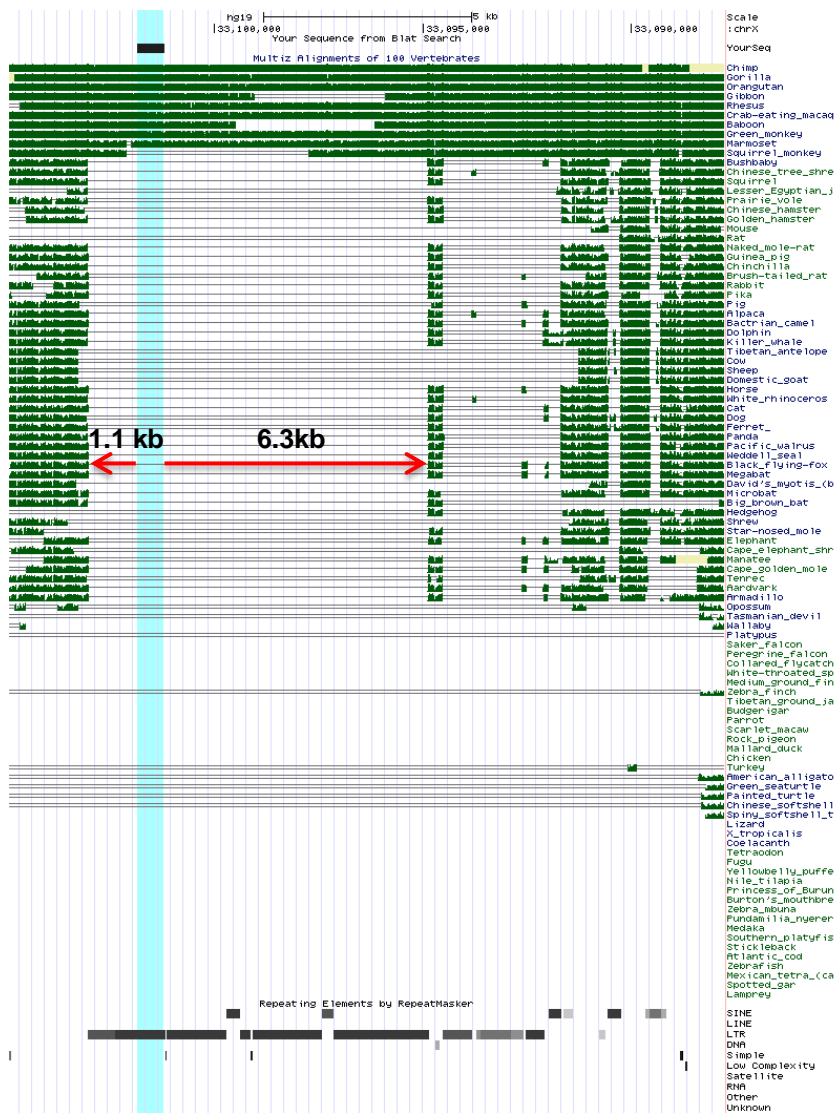

b

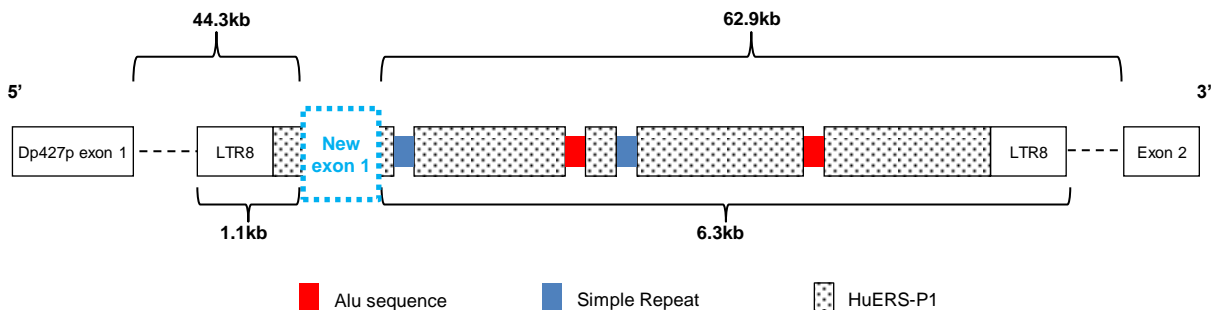

Supplement: Additional file 7: Figure S3. — The new DMD exon 1 belongs to a retrovirus-like sequence. (a) Alignment of the new exon 1 region (highlighted in light blue) among 100 vertebrate species with the conserved upstream/downstream sequence marked by red arrows (https://genome.ucsc.edu). (b) Schematic representation of the approximately 8 kb region in the DMD gene that is conserved among a sub-group of anthropoids. It is composed of simple repeats, Alu sequences and the whole human endogenous retrovirus-like sequence HuERS-P1 (http://www.dfam.org/entry/DF0000214) flanked by two LTR8 elements. [file 13395_2015_62_MOESM7_ESM.pdf]
