# Supplementary material for: Dp412e: a novel human embryonic dystrophin isoform induced by BMP4 in early differentiated cells
Source: Skelet Muscle. 2015 Nov 14;5:40. doi: 10.1186/s13395-015-0062-6 (PMC4644319; doi:10.1186/s13395-015-0062-6)
Supplement: Additional file 6: Figure S2. — 5′RACE reveals the presence of a novel exon 1. (a, b) Two sequences found several times by 5′RACE PCR on RNA from hiPSCs 1 three days after BMP4 treatment. In black are represented the nucleotides found to be spliced to the DMD gene exon 2 by 5′RACE PCR, with in (a), the 164 last nucleotides of Dp427c exon 1 and in (b), the new exon 1. In red are the parts of the RACE sequence corresponding to DMD gene exon 2. The black box in Dp427c sequence points out a nucleotide that does not match with Dp427c exon 1 reference sequence. The BLAT analyses were done on the web site https://genome.ucsc.edu. [file 13395_2015_62_MOESM6_ESM.pdf]

a

Dp427c related RACE sequence (chromosome X position of the sequence in black: 33357376-33357539 (BLAT DNA 2009 (GRCh37/hg19))

GGAATTCAAATAGACTTTCTGGTTCCCAGCAGTCGGCAGTAATAGAATGCTTTCAGGAAGATGACAGAATCAGGAGAAA
GATGCTGTTTTGCACTATCTTGATTTGTTACAGCAGCCAACTTATTGGCATGATGGAGTGACAGGAAAAACAGCTGGCAT
GGAAGATGAAAGAGAAGATGTTCAAAAGAAAACATTTCACAAAATGGGTAANTGCACA

b

New exon 1 related RACE sequence (chromosome X position of the sequence in black corresponding to the new exon 1 : 33101211-33101853 (BLAT DNA 2009 (GRCh37/hg19)), [GenBank: KT072086].

ATACAGAAAATAGGAGGATAGTTTGGAAGGGATACTCTTGGACTTCTTGGTTAGGGATCTGATTTGGAAGGCCTTCTGTC
CCTCTTGTCTTTGCATGTGTTTAAATATGTGAAAGGGATCTCAGAAGGGGTTGCTGATAGAAGTCCAGCATGCCTAACTC
AGAGAACCCTCCTTATTTGTCTGGTCACATTCAGTGAGCTCTAAAGAAGGCTCAACAGTCCTGTCTCTCAGGGTGACTAT
CTGCTCTTTCCCTTGCCCAGAGACCTCATTGTGAATTACCGTTCAGAGGTCATCCGTCCCCACCTGGTGTGGATCAAAGA
CAACAGGGACCAAGATGAAAAATTTTGAGCTTTGCCAGGCTGATATTGGGTGCGGAAAGAGGTGACTAATGTCTGTTTT
GTTATGTGTATTTTGCTGGGTTGGAAAATGTTAATTCAGTTCCCCATGCAGCCCGTTGGGCAGAATCTTGCAAATTAAGA
ATCTTGTTTATGGTTCCATAAAATGGTAAAGGGTGATTTTATCTTGTAAAGTGGCTTAAACCCCACAGCTATAGCACAAG
CAAGCAGGGTCGTAAGACGCCACTCCGTTCTTCTGGAAGCTGCAGAGAAAGGGAACCCAGAAACCTGGCATCCCAGCA
ATAAGGATGAAAGAGAAGATGTTCAAAAGAAAACATTCACAAAATGGGTAAATGCAC
