# Supplementary material for: Dp412e: a novel human embryonic dystrophin isoform induced by BMP4 in early differentiated cells
Source: Skelet Muscle. 2015 Nov 14;5:40. doi: 10.1186/s13395-015-0062-6 (PMC4644319; doi:10.1186/s13395-015-0062-6)
Supplement: Additional file 8: Figure S4. — In silico translation of the novel DMD transcript. Representation of the open reading frame (ORF) resulting in translation of the novel DMD transcript. Possible translated proteins from this ORF are represented in red. The largest predicted protein is composed of 3562 amino acids (≈412 kDa) (http://web.expasy.org/translate). [file 13395_2015_62_MOESM8_ESM.pdf]

I Q K I G G **Stop** F G R D T L G L L G **Stop** G S D L E G L L S L L S L H V F K Y V K G I S E G V A D R S P A C L T Q R T L L I C L V T F S E L
Stop R R L N S P V S Q G D Y L L F P L P R D L I V N Y R S E V I R P H L V W I K D N R D Q D E K F **Stop** A L P G **Stop** Y W V R K E V T N V
C F V **Met** C I L L G W K **Met** L I Q F P **Met** Q P V G Q N L A N **Stop** E S C L W F H K **Met** V K G D F I L **Stop** S G L N P T A I A Q A S R V V R
R H S V L L E A A E K G N P E T W H P S N K D E R E D V Q K K T F T K W V N A Q F S K F G K Q H I E N L F S D L Q D G R R L L D L L E G
L T G Q K L P K E K G S T R V H A L N N V N K A L R V L Q N N N V D L V N I G S T D I V D G N H K L T L G L I W N I I L H W Q V K N V **Met**
K N I **Met** A G L Q Q T N S E K I L L S W V R Q S T R N Y P Q V N V I N F T T S W S D G L A L N A L I H S H R P D L F D W N S V V C Q Q S
A T Q R L E H A F N I A R Y Q L G I E K L L D P E D V D T T Y P D K K S I L **Met** Y I T S L F Q V L P Q Q V S I E A I Q E V E **Met** L P R P P K V
T K E E H F Q L H H Q **Met** H Y S Q Q I T V S L A Q G Y E R T S S P K P R F K S Y A Y T Q A A Y V T T S D P T R S P F P S Q H L E A P E D
K S F G S S L **Met** E S E V N L D R Y Q T A L E E V L S W L L S A E D T L Q A Q G E I S N D V E V V K D Q F H T H E G Y **Met** **Met** D L T A H
Q G R V G N I L Q L G S K L I G T G K L S E D E E T E V Q E Q **Met** N L L N S R W E C L R V A S **Met** E K Q S N L H R V L **Met** D L Q N Q K L
K E L N D W L T K T E E R T R K **Met** E E E P L G P D L E D L K R Q V Q Q H K V L Q E D L E Q E Q V R V N S L T H **Met** V V V V D E S S G
D H A T A A L E E Q L K V L G D R W A N I C R W T E D R W V L L Q D I L L K W Q R L T E E Q C L F S A W L S E K E D A V N K I H T T G F
K D Q N E **Met** L S S L Q K L A V L K A D L E K K K Q S **Met** G K L Y S L K Q D L L S T L K N K S V T Q K T E A W L D N F A R C W D N L V Q
K L E K S T A Q I S Q A V T T T Q P S L T Q T T V **Met** E T V T T V T T R E Q I L V K H A Q E E L P P P P P Q K K R Q I T V D S E I R K R L D
V D I T E L H S W I T R S E A V L Q S P E F A I F R K E G N F S D L K E K V N A I E R E K A E K F R K L Q D A S R S A Q A L V E Q **Met** V N
E G V N A D S I K Q A S E Q L N S R W I E F C Q L L S E R L N W L E Y Q N N I I A F Y N Q L Q Q L E Q **Met** T T T A E N W L K I Q P T T P S
E P T A I K S Q L K I C K D E V N R L S G L Q P Q I E R L K I Q S I A L K E K G Q G P **Met** F L D A D F V A F T N H F K Q V F S D V Q A R E K
E L Q T I F D T L P P **Met** R Y Q E T **Met** S A I R T W V Q Q S E T K L S I P Q L S V T D Y E I **Met** E Q R L G E L Q A L Q S S L Q E Q Q S G L
Y Y L S T T V K E **Met** S K K A P S E I S R K Y Q S E F E E I E G R W K K L S S Q L V E H C Q K L E E Q **Met** N K L R K I Q N H I Q T L K K W
Met A E V D V F L K E E W P A L G D S E I L K K Q L K Q C R L L V S D I Q T I Q P S L N S V N E G G Q K I K N E A E P E F A S R L E T E L
K E L N T Q W D H **Met** C Q Q V Y A R K E A L K G G L E K T V S L Q K D L S E **Met** H E W **Met** T Q A E E E Y L E R D F E Y K T P D E L Q K
A V E E **Met** K R A K E E A Q Q K E A K V K L L T E S V N S V I A Q A P P V A Q E A L K K E L E T L T T N Y Q W L C T R L N G K C K T L E E
V W A C W H E L L S Y L E K A N K W L N E V E F K L K T T E N I P G G A E E I S E V L D S L E N L **Met** R H S E D N P N Q I R I L A Q T L T
D G G V **Met** D E L I N E E L E T F N S R W R E L H E E A V R R Q K L L E Q S I Q S A Q E T E K S L H L I Q E S L T F I D K Q L A A Y I A D K
V D A A Q **Met** P Q E A Q K I Q S D L T S H E I S L E E **Met** K K H N Q G K E A A Q R V L S Q I D V A Q K K L Q D V S **Met** K F R L F Q K P A
N F E Q R L Q E S K **Met** I L D E V K **Met** H L P A L E T K S V E Q E V V Q S Q L N H C V N L Y K S L S E V K S E V E **Met** V I K T G R Q I V Q
K K Q T E N P K E L D E R V T A L K L H Y N E L G A K V T E R K Q Q L E K C L K L S R K **Met** R K E **Met** N V L T E W L A A T D **Met** E L T K
R S A V E G **Met** P S N L D S E V A W G K A T Q K E I E K Q K V H L K S I T E V G E A L K T V L G K K E T L V E D K L S L L N S N W I A V T
S R A E E W L N L L L E Y Q K H **Met** E T F D Q N V D H I T K W I I Q A D T L L D E S E K K K P Q Q K E D V L K R L K A E L N D I R P K V D
S T R D Q A A N L **Met** A N R G D H C R K L V E P Q I S E L N H R F A A I S H R I K T G K A S I P L K E L E Q F N S D I Q K L L E P L E A E I
Q Q G V N L K E E D F N K D **Met** N E D N E G T V K E L L Q R G D N L Q Q R I T D E R K R E E I K I K Q Q L L Q T K H N A L K D L R S Q R
R K K A L E I S H Q W Y Q Y K R Q A D D L L K C L D D I E K K L A S L P E P R D E R K I K E I D R E L Q K K K E E L N A V R R Q A E G L S
E D G A A **Met** A V E P T Q I Q L S K R W R E I E S K F A Q F R R L N F A Q I H T V R E E T **Met** **Met** V **Met** T E D **Met** P L E I S Y V P S T Y L
T E I T H V S Q A L L E V E Q L L N A P D L C A K D F E D L F K Q E E S L K N I K D S L Q Q S S G R I D I I H S K K T A A L Q S A T P V E R
V K L Q E A L S Q L D F Q W E K V N K **Met** Y K D R Q G R F D R S V E K W R R F H Y D I K I F N Q W L T E A E Q F L R K T Q I P E N W E H
A K Y K W Y L K E L Q D G I G Q R Q T V V R T L N A T G E E I I Q Q S S K T D A S I L Q E K L G S L N L R W Q E V C K Q L S D R K K R L E
E Q K N I L S E F Q R D L N E F V L W L E E A D N I A S I P L E P G K E Q Q L K E K L E Q V K L L V E E L P L R Q G I L K Q L N E T G G P V
L V S A P I S P E E Q D K L E N K L K Q T N L Q W I K V S R A L P E K Q G E I E A Q I K D L G Q L E K K L E D L E E Q L N H L L L W L S P I
R N Q L E I Y N Q P N Q E G P F D V Q E T E I A V Q A K Q P D V E E I L S K G Q H L Y K E K P A T Q P V K R K L E D L S S E W K A V N R
L L Q E L R A K Q P D L A P G L T T I G A S P T Q T V T L V T Q P V V T K E T A I S K L E **Met** P S S L **Met** L E V P A L A D F N R A W T E L T
D W L S L L D Q V I K S Q R V **Met** V G D L E D I N E **Met** I I K Q K A T **Met** Q D L E Q R R P Q L E E L I T A A Q N L K N K T S N Q E A R T I I
T D R I E R I Q N Q W D E V Q E H L Q N R R Q Q L N E **Met** L K D S T Q W L E A K E E A E Q V L G Q A R A K L E S W K E G P Y T V D A I
Q K K I T E T K Q L A K D L R Q W Q T N V D V A N D L A L K L L R D Y S A D D T R K V H **Met** I T E N I N A S W R S I H K R V S E R E A A L
E E T H R L L Q Q F P L D L E K F L A W L T E A E T T A N V L Q D A T R K E R L L E D S K G V K E L **Met** K Q W Q D L Q G E I E A H T D V
Y H N L D E N S Q K I L R S L E G S D D A V L L Q R R L D N **Met** N F K W S E L R K K S L N I R S H L E A S S D Q W K R L H L S L Q E L L V
W L Q L K D D E L S R Q A P I G G D F P A V Q K Q N D V H R A F K R E L K T K E P V I **Met** S T L E T V R I F L T E Q P L E G L E K L Y Q E
P R E L P P E E R A Q N V T R L L R K Q A E E V N T E W E K L N L H S A D W Q R K I D E T L E R L Q E L Q E A T D E L D L K L R Q A E V I
K G S W Q P V G D L L I D S L Q D H L E K V K A L R G E I A P L K E N V S H V N D L A R Q L T T L G I Q L S P Y N L S T L E D L N T R W K
L L Q V A V E D R V R Q L H E A H R D F G P A S Q H F L S T S V Q G P W E R A I S P N K V P Y Y I N H E T Q T T C W D H P K **Met** T E L Y
Q S L A D L N N V R F S A Y R T A **Met** K L R R L Q K A L C L D L L S L S A A C D A L D Q H N L K Q N D Q P **Met** D I L Q I I N C L T T I Y D R
L E Q E H N N L V N V P L C V D **Met** C L N W L L N V Y D T G R T G R I R V L S F K T G I I S L C K A H L E D K Y R Y L F K Q V A S S T G F
C D Q R R L G L L L H D S I Q I P R Q L G E V A S F G G S N I E P S V R S C F Q F A N N K P E I E A A L F L D W **Met** R L E P Q S **Met** V W L
P V L H R V A A A E T A K H Q A K C N I C K E C P I I G F R Y R S L K H F N Y D I C Q S C F F S G R V A K G H K **Met** H Y P **Met** V E Y C T P
T T S G E D V R D F A K V L K N K F R T K R Y F A K H P R **Met** G Y L P V Q T V L E G D N **Met** E T P V T L I N F W P V D S A P A S S P Q L
S H D D T H S R I E H Y A S R L A E **Met** E N S N G S Y L N D S I S P N E S I D D E H L L I Q H Y C Q S L N Q D S P L S Q P R S P A Q I L I S
L E S E E R G E L E R I L A D L E E E N R N L Q A E Y D R L K Q Q H E H K G L S P L P S P P E **Met** **Met** P T S P Q S P R D A E L I A E A K L
L R Q H K G R L E A R **Met** Q I L E D H N K Q L E S Q L H R L R Q L L E Q P Q A E A K V N G T T V S S P S T S L Q R S D S S Q P **Met** L L R
V V G S Q T S D S **Met** G E E D L L S P P Q D T S T G L E E V **Met** E Q L N N S F P S S R G R N T P G K P **Met** R E D T **Met** **Stop** E V F S T
W Q **Met** I W A E R W S P **Stop** Y Q S **Stop** Q **Met** K K E Q N K C F T T P D S R **Met** V F I I F I Q Q R G L D S K S L Q E I N L Y F C E G **Stop**
W Y Y T V D F S S F **Stop** V C Y C F V N N G R F Y T S **Met** Q L Y K K V I R K L H V K S **Stop** **Stop** L N N L P F L Y **Met** E R I L G C L K I Y N
S Y K E R L **Stop** T K V C F I K K S C L **Stop** K P L K T K Q T H T H T H T H T H T K L **Stop** G S A L F C I L L A **Stop** Y P Y E I H G F F F F C I
L K I R L P L P P H Q **Met** T T T H C S F E N C Q L S G A G L S F H F I Y L Y V Y K Y I N T I V I **Stop** I K R Y E F L **Stop** T D F F H F L N V H V
T S **Stop** **Stop** K E I T S S Q S S R L T C L V **Stop** N G F F P E P E A R R K L H H T K T L S T A P D V S H F K Q L S T D N E S K V K Y W I F
L K G T C E **Stop** I H R T Y Y I R V S N R L V G **Stop** L I D **Stop** L I H S A S C C **Stop** Q C H D L D L **Met** **Met** L Q W K S I R R Y S D L V N I R
R Y F L T P K Q **Stop** Q D D D R A G G L W I P S P S L **Stop** R S R P L F K **Stop** R I G **Stop** L F I I H K V L C N Y N **Stop** I I **Met** P S S H S Q K
E L G G L V F V A F L D L L S H V G **Stop** V F K C H K T **Stop** F K I N K L W E K V **Stop** N S S P I T F V I L T G I N P E A H E L C F H P L H F
S A S S S T Q V C K **Stop** V R K K A N **Stop** F K C Y K K T L L G G L D R L N I **Stop** T N K Q K L L K K E E K S S R G K A K D W **Stop** E K A L
L F H A I L F L F D F **Stop** I I H S I D T T V **Stop** P I I L Q I C Y L **Stop** H Q V **Stop** L A F G E W A D I K C N **Stop** L L E S G F C P L L I I N **Stop**
L T S N T A S H A I S T S L W F W G V P D N C A H L S S Q L H H L S I A L F S F S F I I L S F S F I I F K R K P K A L R **Stop** Q I T K L H E D L
V F V L H F F P L C D A G P F L Y P R I F K T Q I **Stop** N K G L L Y I L L R S L S K **Stop** V S F **Stop** N Q R **Stop** I E C I N N F V L I F L F F F
Stop T H **Stop** L W S E S V I I F E Q K L R A L L L H F K H N **Stop** F G H Y F V L C S L **Stop** P P S I K L **Stop** I I **Met** **Stop** L K H K H H **Met** A C
F V I V F R Y **Stop** V L T **Stop** V S **Stop** Y I V F **Stop** H Q H C N I Y E L F F **Stop** T S V L L H F H N I S D F T K Y **Met** P Y Y C I I V L L Y C V S
Q **Stop** S T Q L C Y
