# Supplementary material for: Dp412e: a novel human embryonic dystrophin isoform induced by BMP4 in early differentiated cells
Source: Skelet Muscle. 2015 Nov 14;5:40. doi: 10.1186/s13395-015-0062-6 (PMC4644319; doi:10.1186/s13395-015-0062-6)
Supplement: Additional file 9: Figure S7. — Dp412e expression study. Fold change obtained by quantitative RT-PCR using primers specific for Dp427m, Dp427c and Dp412e on RNAs from either human adult tissues, 7–11 weeks old fetuses or human 25–40 weeks old fetal tissues. Gene expression was normalized to UBC and relative to BMP4-treated hiPSCs 1 at day 3. [file 13395_2015_62_MOESM9_ESM.pdf]

Fold change	Dp427m	Dp427c	Dp412e
hiPSCs 1 + BMP4	1	1	1
Fetuses 7-11 weeks	6.16	0.05	1.56E-05
Fetal brain	5.71	4.11	7.95E-07
Brain (whole)	42.79	13.09	2.51E-07
Brain, cerebellum	152.15	15.32	1.89E-07
Brain, cerebral cortex	86.82	33.66	2.56E-07
Thyroid	118.34	0.20	6.16E-07
Trachea	124.03	1.80	5.00E-07
Adrenal	143.24	1.78	5.90E-07
Salivary gland	167.89	0.23	1.22E-06

Fold change	Dp427m	Dp427c	Dp412e
Total heart	958.96	0.05	3.25E-04
Fetal heart	4112.12	0.24	7.49E-07
Lung	105.27	0.09	6.77E-07
Small intestine	505.07	0.53	9.47E-07
Colon	583.13	0.37	1.04E-06
Stomach	1407.58	0.54	1.34E-06
Smooth muscle	482.82	0.47	4.38E-07
Skeletal muscle	2931.32	1.70	4.61E-07
Prostate	919.48	1.62	1.01E-04
Testis	125.70	0.03	2.10E-06
Ovary	771.83	0.09	8.26E-06
Uterus	1215.22	0.47	8.45E-07
